# Inter-kingdom Signaling by the *Legionella* Quorum Sensing Molecule LAI-1 Modulates Cell Migration through an IQGAP1-Cdc42-ARHGEF9-Dependent Pathway

**DOI:** 10.1371/journal.ppat.1005307

**Published:** 2015-12-03

**Authors:** Sylvia Simon, Ursula Schell, Natalie Heuer, Dominik Hager, Michael F. Albers, Jan Matthias, Felix Fahrnbauer, Dirk Trauner, Ludwig Eichinger, Christian Hedberg, Hubert Hilbi

**Affiliations:** 1 Max von Pettenkofer Institute, Ludwig-Maximilians University, Munich, Germany; 2 Institute of Medical Microbiology, University of Zürich, Zürich, Switzerland; 3 Institute of Biochemistry I, University of Cologne, Cologne, Germany; 4 Department of Chemistry, Ludwig-Maximilians University, Munich, Germany; 5 Department of Chemistry and Umeå Center for Microbial Research, Umeå University, Umeå, Sweden; 6 Department of Chemical Biology, Max-Planck-Institute of Molecular Physiology, Dortmund, Germany; Osaka University, JAPAN

## Abstract

Small molecule signaling promotes the communication between bacteria as well as between bacteria and eukaryotes. The opportunistic pathogenic bacterium *Legionella pneumophila* employs LAI-1 (3-hydroxypentadecane-4-one) for bacterial cell-cell communication. LAI-1 is produced and detected by the Lqs (*Legionella* quorum sensing) system, which regulates a variety of processes including natural competence for DNA uptake and pathogen-host cell interactions. In this study, we analyze the role of LAI-1 in inter-kingdom signaling. *L*. *pneumophila* lacking the autoinducer synthase LqsA no longer impeded the migration of infected cells, and the defect was complemented by plasmid-borne *lqsA*. Synthetic LAI-1 dose-dependently inhibited cell migration, without affecting bacterial uptake or cytotoxicity. The forward migration index but not the velocity of LAI-1-treated cells was reduced, and the cell cytoskeleton appeared destabilized. LAI-1-dependent inhibition of cell migration involved the scaffold protein IQGAP1, the small GTPase Cdc42 as well as the Cdc42-specific guanine nucleotide exchange factor ARHGEF9, but not other modulators of Cdc42, or RhoA, Rac1 or Ran GTPase. Upon treatment with LAI-1, Cdc42 was inactivated and IQGAP1 redistributed to the cell cortex regardless of whether Cdc42 was present or not. Furthermore, LAI-1 reversed the inhibition of cell migration by *L*. *pneumophila*, suggesting that the compound and the bacteria antagonistically target host signaling pathway(s). Collectively, the results indicate that the *L*. *pneumophila* quorum sensing compound LAI-1 modulates migration of eukaryotic cells through a signaling pathway involving IQGAP1, Cdc42 and ARHGEF9.

## Author Summary

*Legionella pneumophila* is a ubiquitous environmental bacterium, which upon inhalation causes a severe pneumonia termed Legionnaires’ disease. The opportunistic pathogen employs the small molecule LAI-1 (*Legionella* autoinducer-1) for bacterial cell-cell communication. LAI-1 is produced and detected by the Lqs (*Legionella* quorum sensing) system, which regulates a variety of processes including pathogen-host cell interactions. In this study, we analyzed whether LAI-1 not only plays a role for bacterial signaling but also modulates gene regulation and cellular responses of eukaryotic cells (amoebae or macrophages). We discovered that the gene encoding the LAI-1 autoinducer synthase, *lqsA*, indeed promotes the inhibition of cell migration by *L*. *pneumophila*, and synthetic LAI-1 dose-dependently inhibits cell migration. LAI-1-dependent inhibition of cell migration required the scaffold protein IQGAP1 and the small GTPase Cdc42, as well as the Cdc42 activator ARHGEF9, but not other modulators of Cdc42 or small GTPases. Treatment with LAI-1 led to inactivation of Cdc42 and redistribution of IQGAP1. In summary, our results reveal that the *L*. *pneumophila* signaling compound LAI-1 inhibits the migration of eukaryotic cells through a host signaling pathway comprising IQGAP1, Cdc42 and ARHGEF9.

## Introduction

Bacteria accomplish intra-species and inter-species communication through the production, secretion and detection of low molecular weight compounds [1, 2]. Many of these compounds, termed “autoinducers”, trigger above a certain concentration threshold transmembrane phosphorylation signaling and ultimately gene regulation. The bacterial signaling compounds belong to a variety of chemical classes, including the furanosyl borate ester autoinducer-2 (AI-2), cis-2-dodecenoic acid, alkylhydroxyquinolines (e.g. *Pseudomonas aeruginosa* quinolone signal, PQS), *N*-acylhomoserinelactones (AHLs), or α-hydroxyketones (AHKs) [1–5]. The AHKs CAI-1 (Cholerae autoinducer-1; 3-hydroxytridecane-4-one) and LAI-1 (*Legionella* autoinducer-1; 3-hydroxypentadecane-4-one) have been identified in *Vibrio cholerae* [6] or *Legionella pneumophila* [7] and are produced by the homologous autoinducer synthases CqsA or LqsA, respectively. Moreover, *Janthinobacterium* sp. HH01 [8] and *Photobacterium angustum* [9] harbor CqsA/LqsA orthologues and appear to employ AHK-dependent quorum sensing.

The signaling molecule LAI-1 is produced and sensed by the *lqs* (*Legionella* quorum sensing) genes [10], which are clustered and divergently transcribed from individual promoters [11]. The *lqs* cluster encodes the autoinducer synthase LqsA, the putative cognate sensor kinase LqsS and the prototypic response regulator LqsR [3]. The production of LqsR is dependent on the alternative sigma factor RpoS (σ^38^/σ^S^), and therefore, LqsR is an element of the stationary-phase regulatory network of *L*. *pneumophila* [10]. In addition, the putative sensor kinase LqsT represents an orphan LqsS homolog, which is also a component of the LAI-1 circuit [12]. LqsS and LqsT act as antagonists, as 90% of the genes up-regulated in absence of *lqsS* are down-regulated in absence of *lqsT*. Recent biochemical experiments revealed that LqsS and LqsT are indeed sensor histidine kinases, the auto-phosphorylation of which is regulated by LqsR [13]. In turn, the sensor kinases phosphorylate a conserved aspartate in LqsR, leading to dimerization of the putative response regulator. Synthetic LAI-1 reduces auto-phosphorylation of LqsS and LqsT, regulates gene expression and promotes the motility of *L*. *pneumophila* in the micromolar range [14].

The Lqs system controls pathogen-host cell interactions and production of virulence factors [10, 15]. While *L*. *pneumophila* lacking *lqsA* is only slightly impaired for intracellular replication [16], the *lqsA* mutant strain and all other *lqs* mutants are outcompeted by wild-type bacteria upon co-infection of *Acanthamoeba castellanii* [12]. *L*. *pneumophila* lacking *lqsR* [10], *lqsS* [16], *lqsT* [12] or the whole *lqs* cluster (*lqsA*-*lqsR*-*hdeD*-*lqsS*) [15] are defective for host cell uptake and intracellular replication. The Δ*lqsR* and Δ*lqsS* mutant strains produce a network of extracellular filaments, and therefore, sediment more slowly than wild-type bacteria [16]. Furthermore, in absence of *lqsS*, a 133 kb genomic “fitness island” is up-regulated [16], and all *lqs* mutant strains show much higher natural competence for DNA acquisition [12].

*L*. *pneumophila* is an amoebae-resistant environmental bacterium that can cause a severe pneumonia termed Legionnaires’ disease [17, 18]. The opportunistic pathogen employs the Icm/Dot type IV secretion system (T4SS) and the remarkable number of about 300 different translocated effector proteins to form a replication niche, the *Legionella*-containing vacuole (LCV) and to define other interactions with host cells [19–24]. Accordingly, *L*. *pneumophila* impedes the migration of infected *Dictyostelium discoideum* amoebae and mammalian cells in an Icm/Dot-dependent manner [25]. The Icm/Dot-translocated effector protein LegG1, a Ran GTPase activator [26], antagonizes migration inhibition by Ran-dependent microtubule stabilization.

The small GTPases RhoA, Rac1 and Cdc42 promote directional migration, proper microtubule assembly and actin cytoskeleton organization in the cell, in concert with the scaffold protein IQGAP1, which represents a key node within the small GTPase network [27]. In the present study, we show that the *L*. *pneumophila* quorum sensing compound LAI-1 inhibits cell migration through a signaling pathway involving IQGAP1, Cdc42 and the Cdc42 activator ARHGEF9.

## Results

### Effect of *L*. *pneumophila lqs* genes on host cell migration

**Fig 1:**
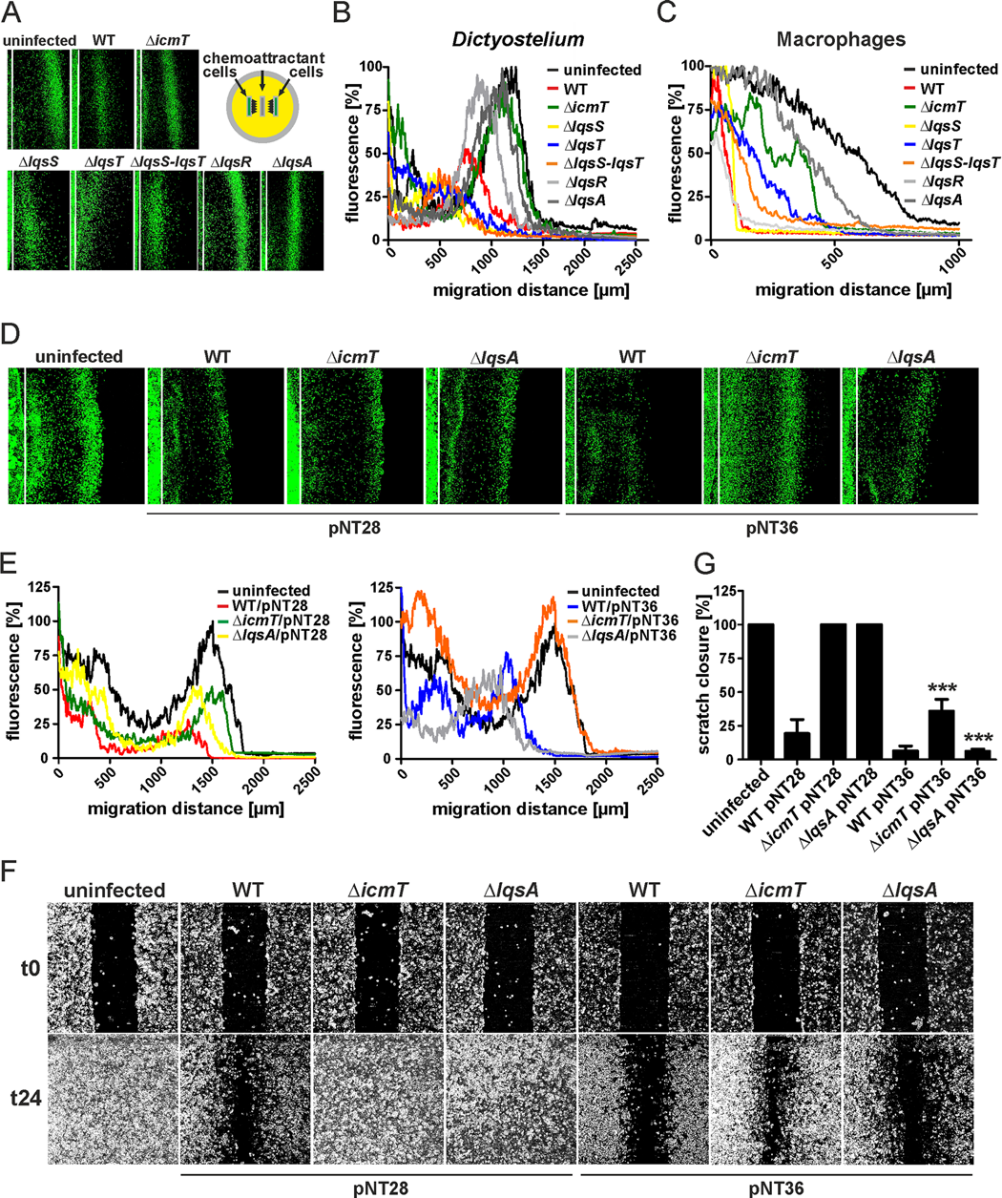
Effect of *L*. *pneumophila lqs* genes on host cell migration. *D*. *discoideum* strain Ax3 producing GFP (pSW102) was infected (MOI 10, 1 h) with (A) *L*. *pneumophila* wild-type, Δ*icmT*, Δ*lqsS*, Δ*lqsT*, *ΔlqsS-lqsT*, Δ*lqsR* or Δ*lqsA* mutant strains harboring pSW001 (DsRed), or with (D) the strains harboring pNT28 (GFP) or pNT36 (GFP, LqsA). An under-agarose assay was used to monitor the migration towards folate (1 mM) for another 4 h. The white lines represent the edge of the sample wells. (B, E) Graphs of the data from (A, D) plotted as per cent GFP fluorescence intensity versus migration distance. (C) Murine RAWs 264.7 macrophages were infected (MOI 10, 1 h) with *L*. *pneumophila* wild-type, Δ*icmT*, Δ*lqsS*, Δ*lqsT*, *ΔlqsS-lqsT*, Δ*lqsR* or Δ*lqsA* mutant strains. Cells were stained with Cell Tracker Green BODIPY and let migrate towards CCL5 (100 ng/ml) in an under-agarose assay for another 4 h. Graphs show the per cent fluorescence intensity versus migration distance. (F) Confluent cell layers of A549 epithelial cells were left uninfected or infected (MOI 10, 1 h) with *L*. *pneumophila* wild-type, Δ*icmT* or Δ*lqsA* mutant strains harboring pNT28 (GFP) or pNT36 (GFP, LqsA), scratched and let migrate for 24 h. Prior to imaging (0, 24 h), the detached cells were washed off. (G) The scratch area was quantified using ImageJ software at 7 different positions per condition in triplicate samples. Means and standard deviations of the triplicate samples are shown (pNT28 vs. pNT36: ***p < 0.001). The data shown are representative of at least 3 independent experiments.

Wild-type *L*. *pneumophila*, but not mutant bacteria lacking a functional Icm/Dot T4SS, inhibit cell migration of free-living amoebae and mammalian cells [25]. To further define the bacterial factors implicated in inhibition of cell migration, we infected *D*. *discoideum* amoebae or RAW 264.7 macrophages with *L*. *pneumophila* mutant strains lacking components of the Lqs quorum sensing system and tested the effects on cell migration in under-agarose chemotaxis assays (Fig 1A). *L*. *pneumophila* strains lacking *lqsR*, *lqsS* and/or *lqsT* inhibited the chemotactic migration of *D*. *discoideum* (Fig 1B) or macrophages (Fig 1C) to a similar extent as wild-type bacteria, suggesting that these Lqs components, albeit implicated in bacterial virulence, play a minor role for cell migration inhibition. In contrast, however, the migration of *D*. *discoideum* or macrophages infected with *L*. *pneumophila* Δ*lqsA* was not inhibited (similar to cells infected with Δ*icmT* mutant bacteria).

Next, we tested in the *D*. *discoideum* under-agarose assay the effects of over-expressing *lqsA* in the Δ*lqsA* or Δ*icmT* mutant strains or in wild-type *L*. *pneumophila* (Fig 1D). The overexpression of plasmid-borne *lqsA* partially complemented the defect in migration inhibition of *D*. *discoideum* by the Δ*lqsA* mutant strain (Fig 1E). To analyze the effects of over-expressing *lqsA* in another cell migration system, we used A549 lung epithelial carcinoma cells and a scratch wound healing assay [25] (Fig 1F). Under these conditions, the overexpression of *lqsA* in the Δ*lqsA* mutant strain completely restored the inhibition of cell migration (Fig 1G). Moreover, the overexpression of *lqsA* in the Δ*icmT* mutant strain significantly inhibited A549 cell migration, suggesting that LqsA (and in consequence, LAI-1) inhibit the migration of eukaryotic cells. Taken together, while most *lqs* genes do not appear to play a major role for cell migration inhibition by *L*. *pneumophila*, *lqsA* is required for inhibiting the migration of amoebae, macrophages and epithelial cells. These findings suggest that the signaling molecule LAI-1 produced by the autoinducer synthase LqsA might directly or indirectly affect cell migration.

### Dose-dependent inhibition of chemotaxis and cell migration by LAI-1

**Fig 2:**
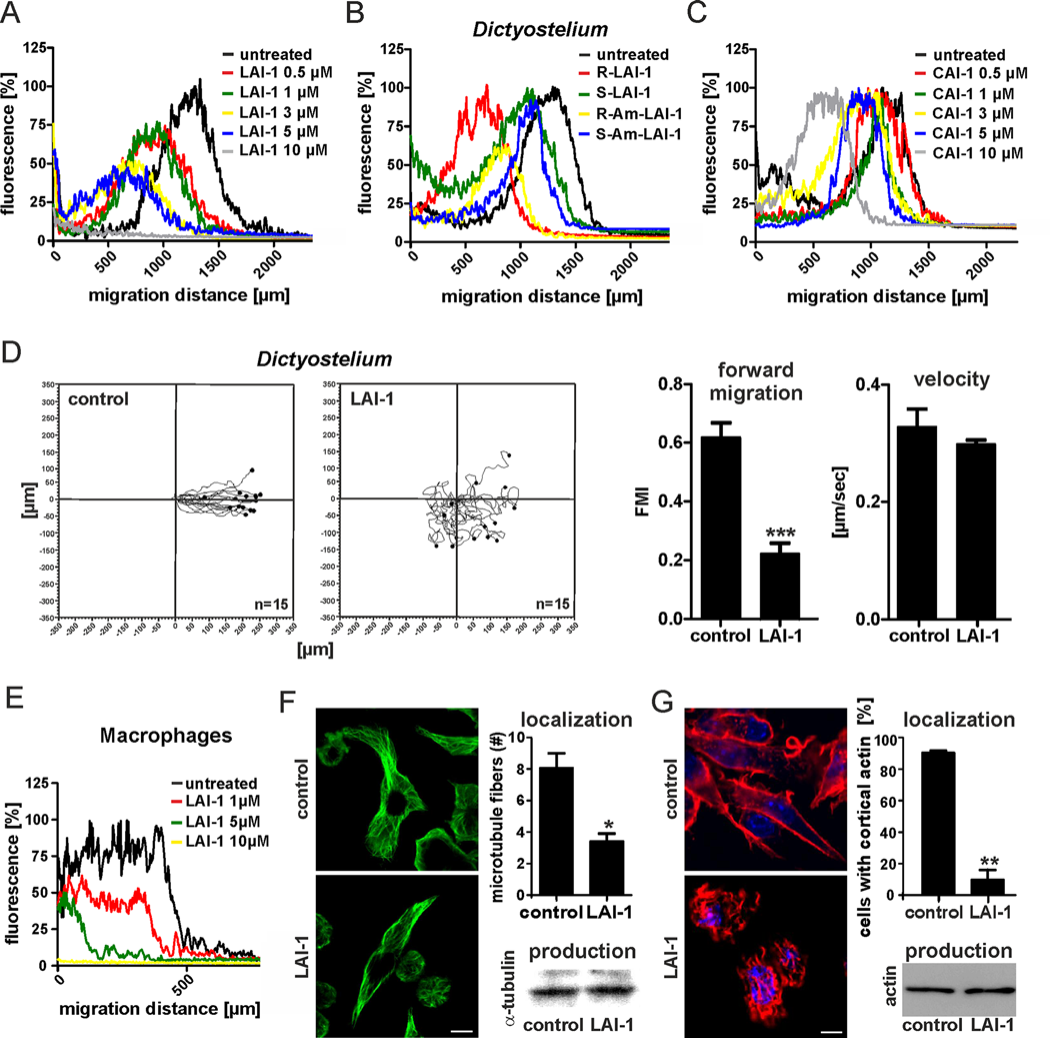
Dose-dependent inhibition of chemotaxis and cell migration by LAI-1. *D*. *discoideum* amoebae harboring pSW102 (GFP) were treated for 1 h with different concentrations of (A) racemic LAI-1, (B) 10 μM (*R*)-LAI-1, (*S*)-LAI-1, (*R*)-amino-LAI-1 or (*S*)-amino-LAI-1, or (C) different concentrations of CAI-1, and cell migration towards folate (1 mM) was monitored in under-agarose assays for 4 h. Graphs depict per cent GFP fluorescence intensity versus migration distance. (D) *D*. *discoideum* amoebae harboring pSW102 (GFP) were treated with LAI-1 (10 μM, 1 h). Single cell migration towards folate (1 mM) was monitored in under-agarose assays for 15 min. Motility parameters (forward migration index, FMI; and velocity) were analyzed using the ImageJ manual tracker and Ibidi chemotaxis software. (E) Murine RAW 264.7 macrophages were treated for 1 h with different concentrations of racemic LAI-1, cell migration towards CCL5 (100 ng/ml) was monitored in under-agarose assays for 4 h, and the cells were stained with Cell Tracker Green BODIPY. Macrophages treated for 1 h with 10 μM LAI-1 were immuno-labeled for (F) α-tubulin (green) or (G) actin (red) and, as a control, the production of cellular α-tubulin or actin was quantified by Western blot. Microtubule fibers per cell were counted along cross-sections (S3 Fig), and the actin architecture was analyzed by quantifying the number of cells displaying cortical actin. The graphs show means and standard deviations of 3 independent experiments (n > 25 (α-tubulin) or > 40 (actin) single cells; Student´s t-test, *p < 0.05, **p < 0.01). Bars (F, G), 5 μm.

Based on the genetic results, we tested whether synthetic LAI-1 modulates chemotaxis and migration of eukaryotic cells. To this end, *D*. *discoideum* amoebae were treated for 1 h with different concentrations of LAI-1 and cell migration towards folate was monitored in under-agarose assays for 4 h (Fig 2A). We observed that 0.5–10 μM racemic LAI-1 inhibited migration of the amoebae in a dose-dependent manner.

The biosynthetic pathway and the stereochemistry of LAI-1 are currently unknown. Given the efficacy of LAI-1-meditated inhibition of cell migration, we sought to employ this readout to assess the biological activity of enantiomers of LAI-1 and its putative precursor amino-LAI-1 [28, 29]. We treated *D*. *discoideum* with 10 μM (*R*)- or (*S*)-LAI-1, and with 10 μM (*R*)- or (*S*)-amino-LAI-1. The (*R*)-enantiomers of LAI-1 or amino-LAI-1 inhibited the migration of the amoebae more efficiently than the (*S*)-enantiomers (Fig 2B). Furthermore, the *V*. *cholerae* signaling molecule CAI-1 also impeded the migration of *D*. *discoideum* in under-agarose chemotaxis assays: 0.5–10 μM CAI-1 inhibited migration of the amoebae in a dose-dependent manner (Fig 2C). Neither LAI-1 nor CAI-1 acted as chemo-attractants, and up to 50 μM LAI-1 did not affect uptake or cytotoxicity of *L*. *pneumophila* in *D*. *discoideum* (S1 Fig).

We also investigated as motility parameters the forward migration index and the velocity of *D*. *discoideum* amoebae treated with 10 μM LAI-1 (Fig 2D). Single cell tracking analysis using the ImageJ manual tracker and Ibidi chemotaxis software revealed that upon treatment with LAI-1 the forward migration index was reduced by approximately 50%, while the velocity of the amoebae was not affected. These results indicate that the directionality but not the speed of the phagocytes was impaired by the bacterial quorum sensing signal. Taken together, the chemotactic migration of *D*. *discoideum* amoebae was found to be inhibited in a dose-dependent manner by LAI-1 or CAI-1, and the (*R*)-enantiomers of the α-hydroxyketones or α-aminoketones are biologically more active with respect to inhibition of cell migration.

To assess whether the migration of macrophages is also affected by LAI-1, we treated murine macrophage-like RAW 264.7 cells with different concentrations of racemic LAI-1 and monitored cell migration towards the chemokine CCL5 in under-agarose assays for 4 h (Fig 2E). Similar to amoebae, migration of macrophages was also inhibited in a dose-dependent manner upon treatment with 1–10 μM LAI-1. Also, up to 10 μM LAI-1 did not affect uptake or cytotoxicity of *L*. *pneumophila* in macrophages (S1 Fig).

Next, we tested the effects of LAI-1 on the cellular microtubule and actin cytoskeleton. RAW 264.7 macrophages were treated with 10 μM LAI-1, the cells were then immuno-labeled for α-tubulin, and microtubule polymerization was quantified by counting the number of microtubule fibers along cross-sections (Figs 2F and S2A). These experiments revealed that LAI-1 reduced the number of microtubule fibers per cell by approximately 50%, while the overall amount of α-tubulin was not affected. As a control for microtubule disintegration, the cells were treated with 30 μM of the microtubule destabilizing compound nocodazole.

Analogously, we visualized the actin network by fluorescence microscopy in macrophages exposed to 10 μM LAI-1 (Fig 2G). The treatment with LAI-1 markedly altered the architecture of the actin cytoskeleton. We found that the cortical actin nearly completely disappeared upon treatment with the *L*. *pneumophila* signaling molecule. At the same time, treatment with LAI-1 did not affect the overall amount of actin in the cell, as revealed by Western blot (Fig 2G). Taken together, LAI-1 inhibits cell migration by profoundly affecting microtubule polymerization as well as the F-actin network architecture.

### LAI-1-dependent inhibition of cell migration requires IQGAP1 and Cdc42

**Fig 3:**
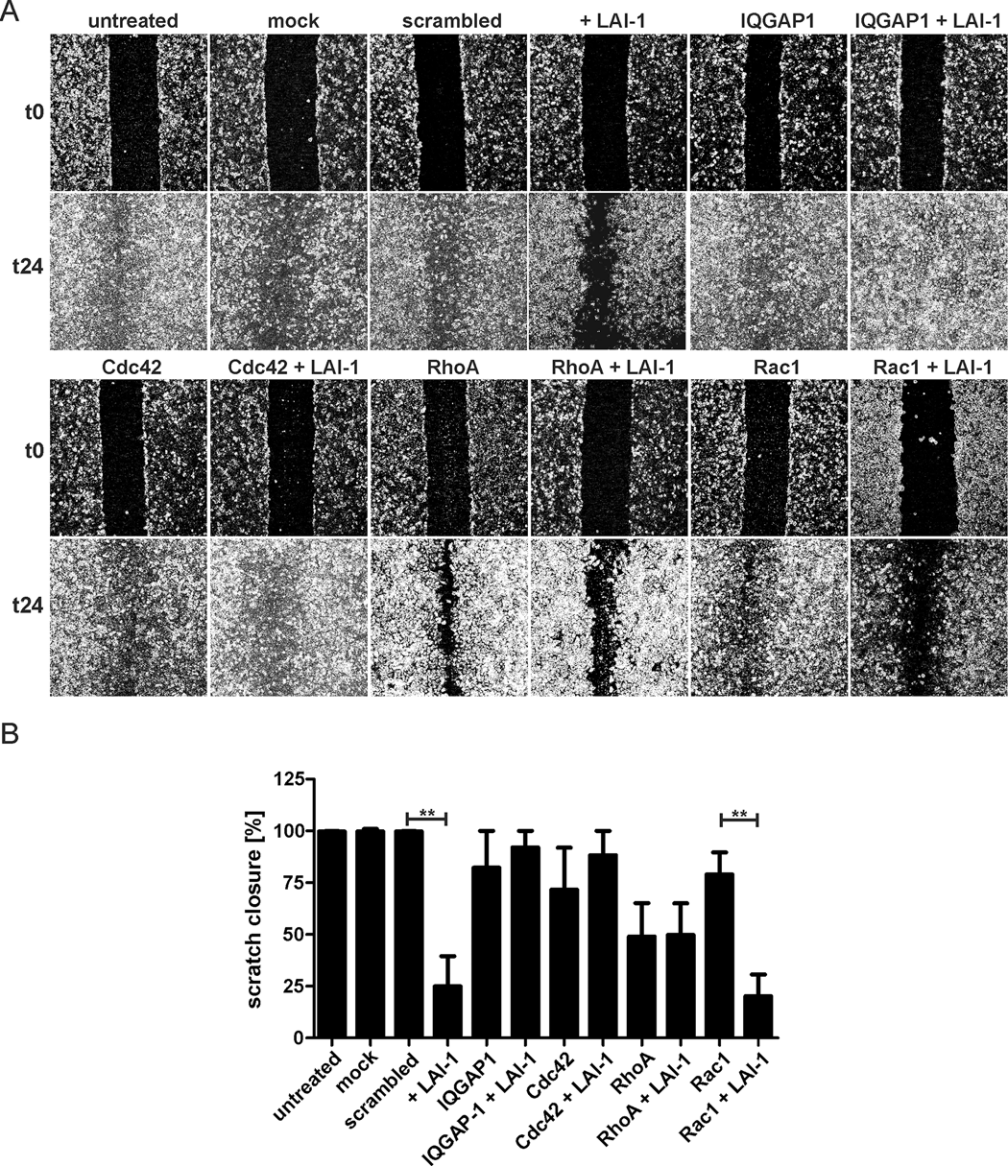
LAI-1-dependent inhibition of cell migration requires IQGAP1 and Cdc42. (A) Confluent cell layers of A549 epithelial cells were treated with siRNA against IQGAP1, Cdc42, RhoA or Rac1 for 2 days. The cells were then treated or not with LAI-1 (10 μM, 1.5 h), scratched and let migrate for 24 h. Prior to imaging (0, 24 h), the detached cells were washed off. (B) The scratch area was quantified using ImageJ software at 7 different positions per condition in triplicate samples. Means and standard deviations of triplicate samples are shown (**p < 0.01). The data is representative of 3 independent experiments.

LAI-1 inhibits the chemotactic migration of phagocytes towards folate or CCL5 (Fig 2). To test whether LAI-1 also inhibits migration of eukaryotic cells in absence of an exogenously added chemo-attractant, we used the A549 lung epithelial cells and the scratch wound healing assay (Figs 3 and S2B). Confluent layers of the epithelial cells were treated or not with 10 μM LAI-1, scratched and let migrate for 24 h. Within this period of time, untreated cells repopulated the scratch area and thus formed a confluent layer again. In contrast, cells treated with 10 μM LAI-1 were severely impaired for migration (Fig 3A), and the area of the scratch was closed to only 25% (Fig 3B). Thus, LAI-1 not only inhibits directed migration towards an exogenously added chemo-attractant but also towards a scratch wound.

A549 cells are readily amenable to RNA interference (RNAi), thus allowing the assessment of host factors during *L*. *pneumophila* infection [25, 26, 30]. Analogously, factors implicated in LAI-1-dependent signaling can be investigated. To assess possible eukaryotic factors involved in LAI-1-dependent signaling, we studied the roles of the scaffold protein IQGAP1 and small GTPases of the Rho/Rac/Cdc42 family implicated in cytoskeletal dynamics [31, 32]. IQGAP1, Cdc42, RhoA or Rac1 were depleted by RNAi for 2 days in confluent layers of A549 cells, which were then treated or not with 10 μM LAI-1, scratched and let migrate for another 24 h (Fig 3A). Western blot analysis revealed that after the RNAi treatment the proteins were not detectable anymore (S3 Fig). Upon depletion of IQGAP1 or Cdc42 (but not RhoA or Rac1) LAI-1 no longer prevented scratch closure compared to cells treated only with the corresponding siRNA (Fig 3B). Therefore, IQGAP1 and Cdc42, but not RhoA or Rac1, promote the transmission of LAI-1-mediated inter-kingdom signaling. The depletion of IQGAP1, Cdc42 or Rac1 did not significantly affect the scratch closure of untreated cells, yet the depletion of RhoA reduced scratch closure by approximately 50%, regardless of whether LAI-1 was present or not (Fig 3B).

The Icm/Dot-translocated Ran activator LegG1 antagonizes the inhibition of cell migration by *L*. *pneumophila* in a Ran GTPase-dependent manner [25]. To assess a role for Ran and its effector RanBP1 in LAI-1-mediated inhibition of cell migration, we depleted the small GTPase or its effector in A549 cells and performed scratch assays upon treatment with LAI-1 (S4A Fig). In these experiments, neither Ran nor RanBP1 were found to play a significant role in LAI-1-mediated inhibition of cell migration.

### LAI-1 promotes inactivation of Cdc42 and redistribution of IQGAP1 to the cell cortex

**Fig 4:**
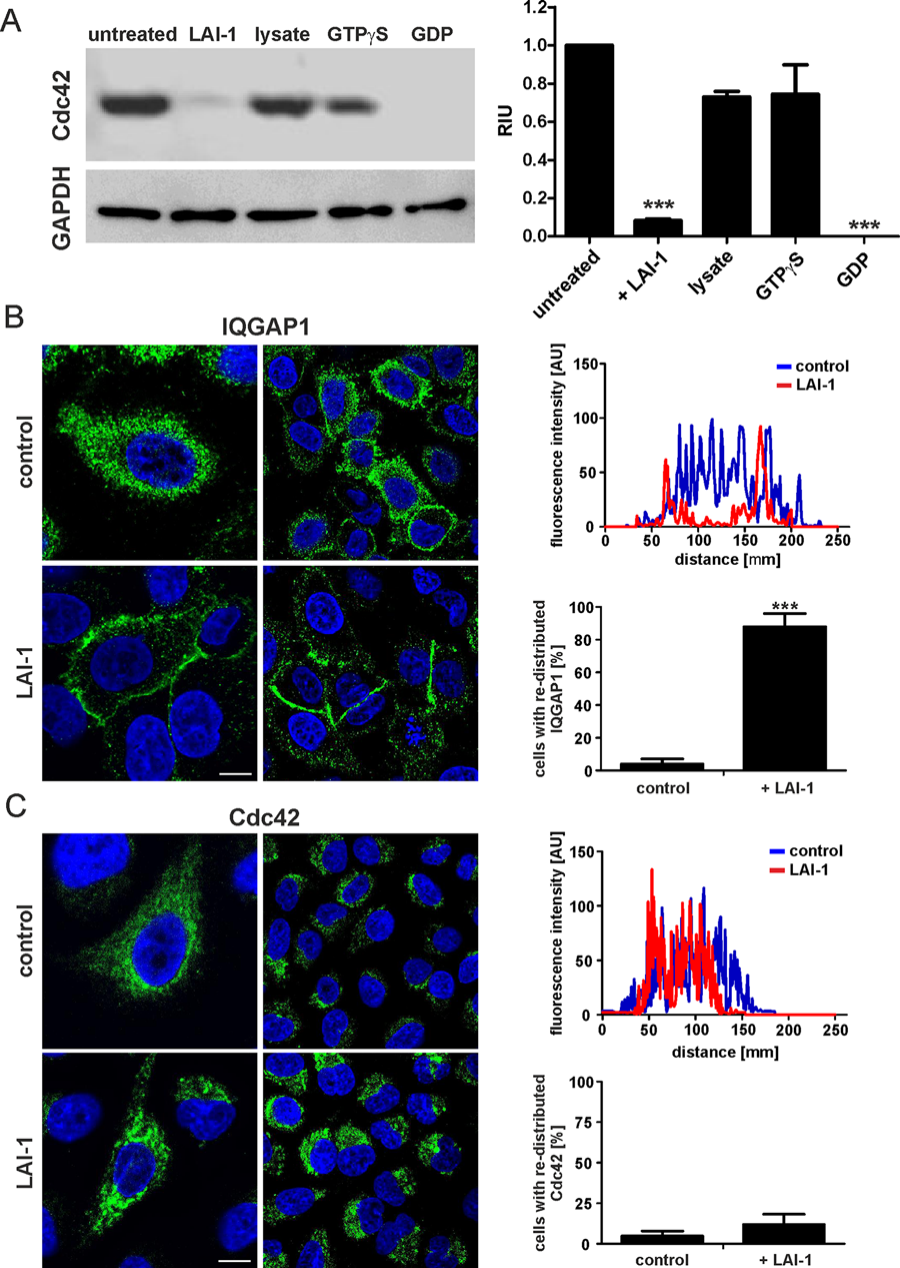
LAI-1 promotes inactivation of Cdc42 and redistribution of IQGAP1 to the cell cortex. (A) A549 cells were treated with LAI-1 (10 μM, 1 h), and the activation state of Cdc42 was analyzed by Western blot using an antibody recognizing Cdc42(GTP/GDP) (left panel). Quantification by densitometry was performed using ImageJ (right panel). A549 cells were treated with LAI-1 (10 μM, 1 h), fixed, stained with antibodies against (B) IQGAP1 or (C) Cdc42 and analyzed by confocal microscopy (left panels; green, FITC; blue, DAPI). The graphs (right panels) are based on the relative fluorescence intensity along cell sections (n = 50, ***p < 0.001) and show that upon LAI-1 treatment IQGAP1 but not Cdc42 redistributes to the cell cortex. Bars (B, C), 5 μm.

To investigate whether LAI-1 modulates the activation of Cdc42, a pulldown assay was performed. A549 cells treated or not with 10 μM LAI-1 for 1 h were lysed, and the lysate was incubated with GST-PBD_Pak1_, a fusion protein specifically binding activated Cdc42. After pulldown with glutathione resin, the amount of active Cdc42(GTP) was quantified by densitometry of Western blots using an antibody that specifically recognizes Cdc42(GTP) (Fig 4A). The analysis revealed that upon treatment of the cells with LAI-1 the concentration of active Cdc42(GTP) decreased approximately 10-fold. As an input control, the amount of GAPDH in the cell lysates was determined by Western blot. These findings were confirmed by an analogous approach using an anti-Cdc42(GTP/GDP) antibody instead of GST-PBD_Pak1_, followed by Western blot with an antibody recognizing Cdc42(GTP) (S5A Fig). Using an antibody that recognizes Cdc42/Rac1-phospho-Ser71, we also assessed the phosphorylation state of Cdc42 in response to LAI-1 by Western blot (S5B Fig) or fluorescence microscopy (S5C Fig). Yet, we did not observe changes in the phosphorylation pattern or intensity upon treatment of the cells with LAI-1. Taken together, LAI-1 signaling promotes the inactivation of Cdc42, without affecting the phosphorylation of the small GTPase.

To analyze whether LAI-1 alters the spatial distribution of the scaffold protein IQGAP1 or the small GTPase Cdc42, we incubated A549 cells for 1 h with 10 μM of the quorum sensing compound and stained the cells with an antibody against IQGAP1 (Fig 4B) or Cdc42 (Fig 4C). Upon treatment of the cells with LAI-1, IQGAP1 redistributed from the cytoplasm to the cell cortex. The re-localization was very efficient, as nearly 100% of the cells observed by microscopy showed a cortical accumulation of IQGAP1 after exposure to LAI-1. In contrast, the cytoplasmic localization of Cdc42 remained unaffected by LAI-1 treatment. The treatment with LAI-1 did not affect the overall amount of IQGAP1, Cdc42 or Rac1 (S5D Fig).

### LAI-1-dependent redistribution of IQGAP1 does not require Cdc42

**Fig 5:**
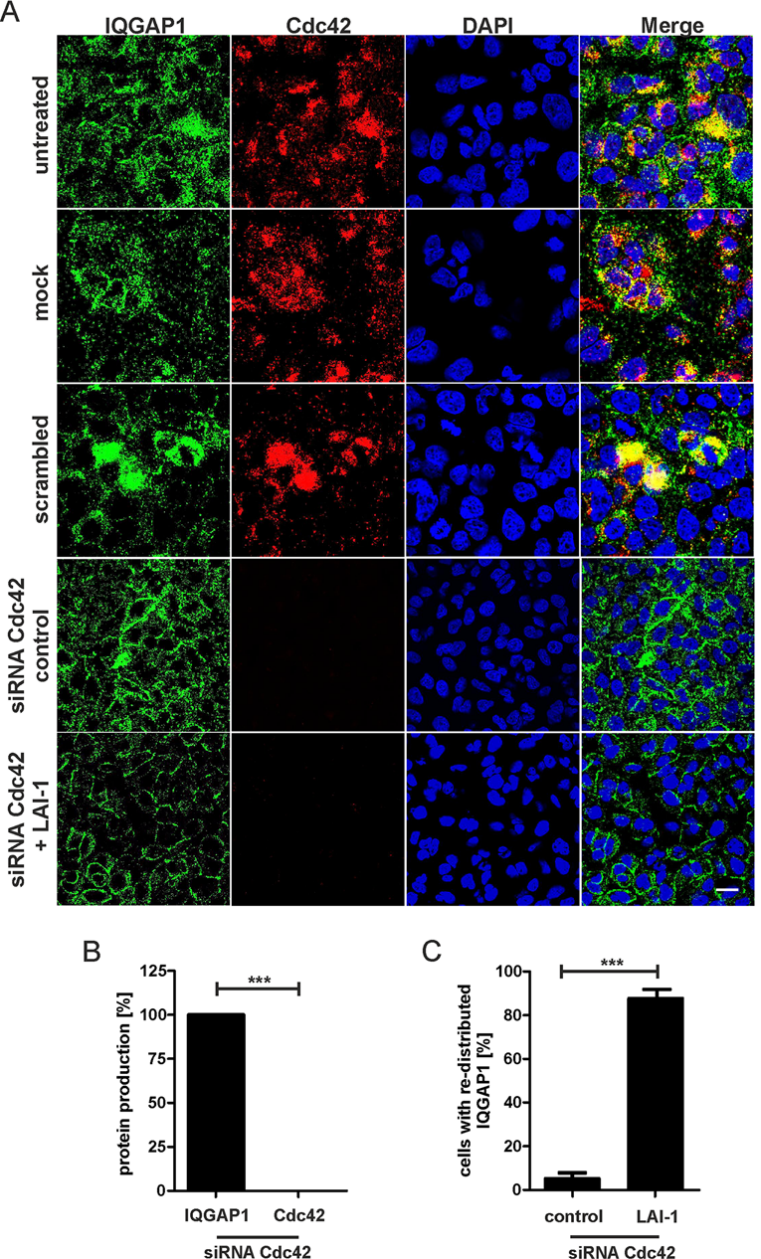
LAI-1-dependent redistribution of IQGAP1 does not require Cdc42. (A) A549 cells were treated with siRNA against Cdc42 for 2 days, then with LAI-1 (10 μM, 1.5 h) and protein production or the subcellular localization of the IQGAP1 scaffold protein (green) and the small GTPase Cdc42 (red) was analyzed by confocal microscopy using antibodies against IQGAP1 or Cdc42. Nuclei were stained with DAPI (blue). (B) Quantification of protein production in A549 cells after RNAi treatment (percent cells producing protein of interest; n = 50). Means and standard deviations of 3 independent experiments are shown (***p < 0.001). (C) Redistribution of IQGAP1 in absence of Cdc42 upon LAI-1 treatment. The graph is based on the relative fluorescence intensity along cell sections (n = 50). Means and standard deviations of 3 independent experiments are shown (***p < 0.001). Bar, 20 μm.

To test whether in the LAI-1 signal transduction pathway the redistribution of IQGAP1 to the cell cortex requires Cdc42, we depleted Cdc42 in A549 cells, followed by exposure to 10 μM LAI-1 and an analysis of the cellular localization of IQGAP1 by fluorescence microscopy (Fig 5A). Treatment of the cells with siRNA against Cdc42 efficiently depleted the cells of the small GTPase (Figs 5B and S3). Under these conditions, IQGAP1 still re-distributed to the cell cortex upon treatment with LAI-1, indicating that Cdc42 is dispensable for the re-localization of the scaffold protein (Fig 5C). Thus, treatment of A549 cells with LAI-1 leads to the deactivation of the small GTPase Cdc42, as well as to the redistribution to the cell cortex of the scaffold protein IQGAP1, which is located upstream of Cdc42 in the LAI-1 signaling cascade.

### LAI-1-dependent inhibition of cell migration requires the Cdc42 GEF ARHGEF9

**Fig 6:**
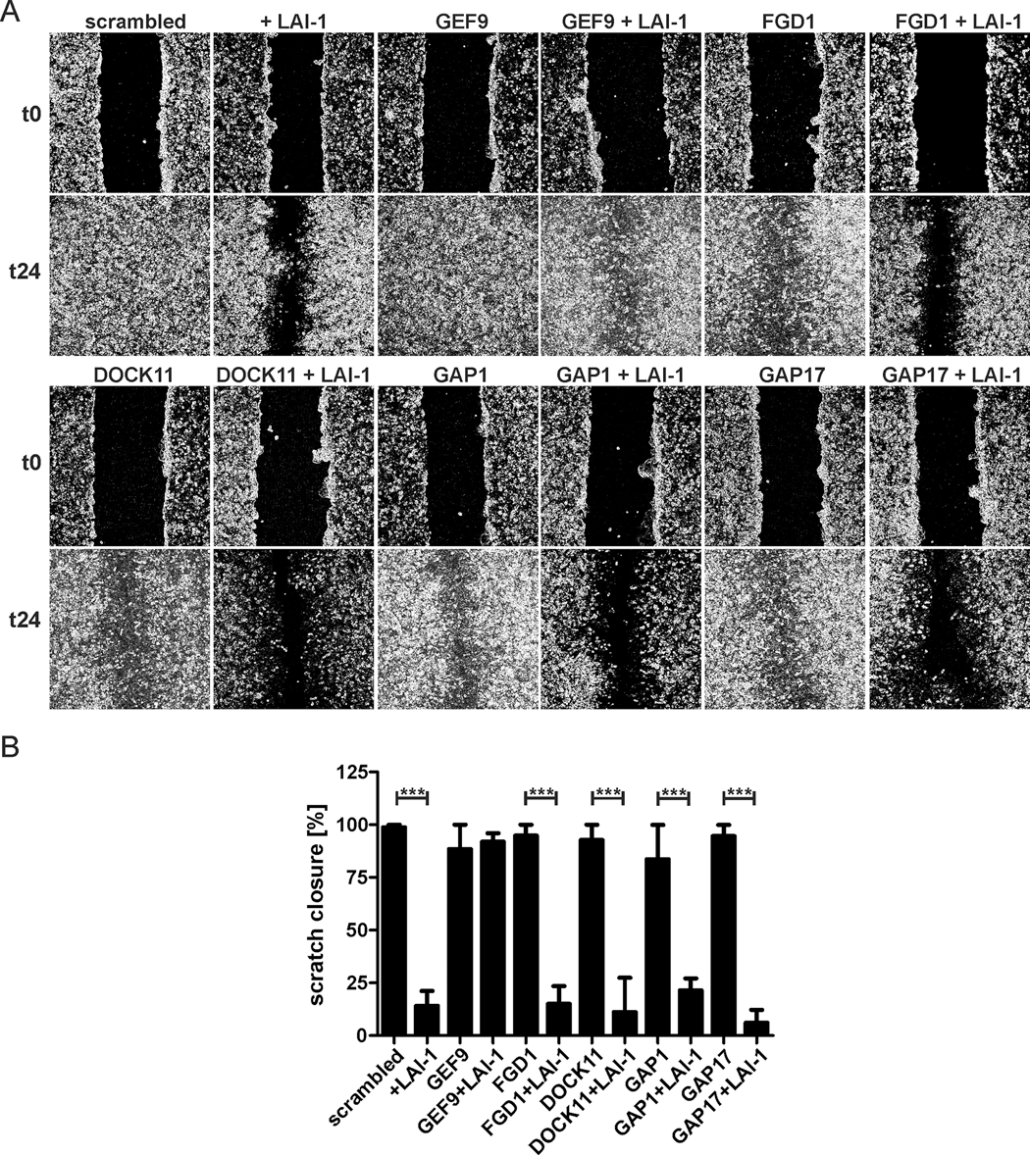
LAI-1-dependent inhibition of cell migration requires the Cdc42 GEF ARHGEF9. (A) Confluent cell layers of A549 cells were treated for 2 days with siRNA against the different Cdc42 GEFs or GAPs indicated. The cells were then treated or not with LAI-1 (10 μM, 1.5 h), scratched and let migrate for 24 h. Prior to imaging (0, 24 h), the detached cells were washed off. (B) The scratch area was quantified at 6 different positions per condition using ImageJ software. Means and standard deviations of 3 samples are shown, which are representative of 3 independent experiments (***p < 0.001).

In order to obtain further insight into the LAI-1-dependent inactivation of Cdc42, we depleted by RNAi different guanine nucleotide exchange factors (GEFs) or GTPase activating proteins (GAPs) specific for Cdc42. Confluent cell layers of A549 cells were treated with siRNA against the Cdc42-specific GEFs ARHGEF9, FGD1 or DOCK11, or against GAPs with more relaxed specificity, ARHGAP1 or ARHGAP17 (Fig 6A). Western blot analysis revealed that after the RNAi treatment the proteins were not detectable anymore (S3 Fig). Among these GTPase modulators, only ARHGEF9 appeared to be significantly involved in LAI-1-mediated cell migration inhibition, as the depletion of this GEF abolished the inhibitory action of LAI-1 (Fig 6B).

### The scaffold protein CD2AP is not involved in LAI-1-mediated migration inhibition

In an attempt to identify other host factors possibly implicated in LAI-1-mediated inhibition of cell migration, the cellular transcriptome of *D*. *discoideum* was analyzed in response to the bacterial signaling molecule. We exposed exponentially growing *D*. *discoideum* to 20 μM of synthetic LAI-1 for 3 h and compared gene regulation by transcriptome analysis to untreated amoebae. Under these conditions, LAI-1 was found to up- or down-regulate 115 or 144 genes respectively, at least 1.5-fold (S6A Fig, S1 and S2 Tables). This number of genes constitutes approximately 5% of the 5,400 genes on the array [33]. 74 up- and 113 down-regulated genes, respectively, could be functionally categorized based on the yeast classification scheme which was adapted for *Dictyostelium* [34]. Notably, in the categories “protein destination” (including vesicle trafficking), and in “signal transduction” most differentially regulated genes were up-regulated. In contrast, in the categories “translation”, “cell proliferation” and “movement” most of the genes were down-regulated (S6A Fig). The latter result is in agreement with the notion that treatment with LAI-1 directly or indirectly may modulate (reduce) cell movement.

Several genes of the ubiquitin proteasome system, the “core” autophagy genes *atg8* and *atg16* as well as the autophagy adaptor sequestosome-1 were up-regulated. In addition, we noted three members of the ABC transporter G family, a gene named *iliA* (induced after *L* *egionella* infection) and the gene DDB_G0274423 which encodes a Src homology 3 (SH3) domain-containing protein (S1 and S2 Tables). The latter gene is homologous to CD2AP, a scaffold protein modulating actin dynamics and cell migration [35]. Down-regulated genes include the aldehyde reductases *alrA* and *alrE*, several genes of the counting factor complex, the putative metallophosphoesterase *dduA*, *rliA* (repressed after *L* *egionella* infection) encoding a putative 12 transmembrane domain protein of the major facilitator family, as well as several peptidase encoding genes (S1 and S2 Tables). The regulation of six of these genes (three up- and three down-regulated) was validated by quantitative real time (RT)-PCR (S6B Fig). In summary, whole-genome transcriptome analysis revealed that synthetic LAI-1 in the micromolar concentration range regulates the expression of a number of eukaryotic genes involved in different processes, including protein homeostasis, vesicle trafficking, and cell migration.

Based on the finding that LAI-1 increased the expression of *D*. *discoideum* DDB_G0274423 encoding a Src homology 3 (SH3) domain-containing protein, we sought to analyze its potential role in LAI-1-mediated inhibition of cell migration. We depleted by RNAi for 2 days its mammalian homologue, CD2AP, in confluent layers of A549 cells, which were then treated or not with 10 μM LAI-1, scratched and let migrate for another 24 h (S4B Fig). Western blot analysis revealed that after the RNAi treatment CD2AP was not detectable anymore (S3 Fig). Yet, compared to untreated cells or cells treated with scrambled siRNA oligonucleotides or mock-treated cells, the depletion of CD2AP had no effect on LAI-1-mediated inhibition of cell migration (S4C Fig). Therefore, CD2AP appears to be dispensable for LAI-1-dependent signaling.

### LAI-1 reverses Icm/Dot-dependent migration inhibition by *L*. *pneumophila*

**Fig 7:**
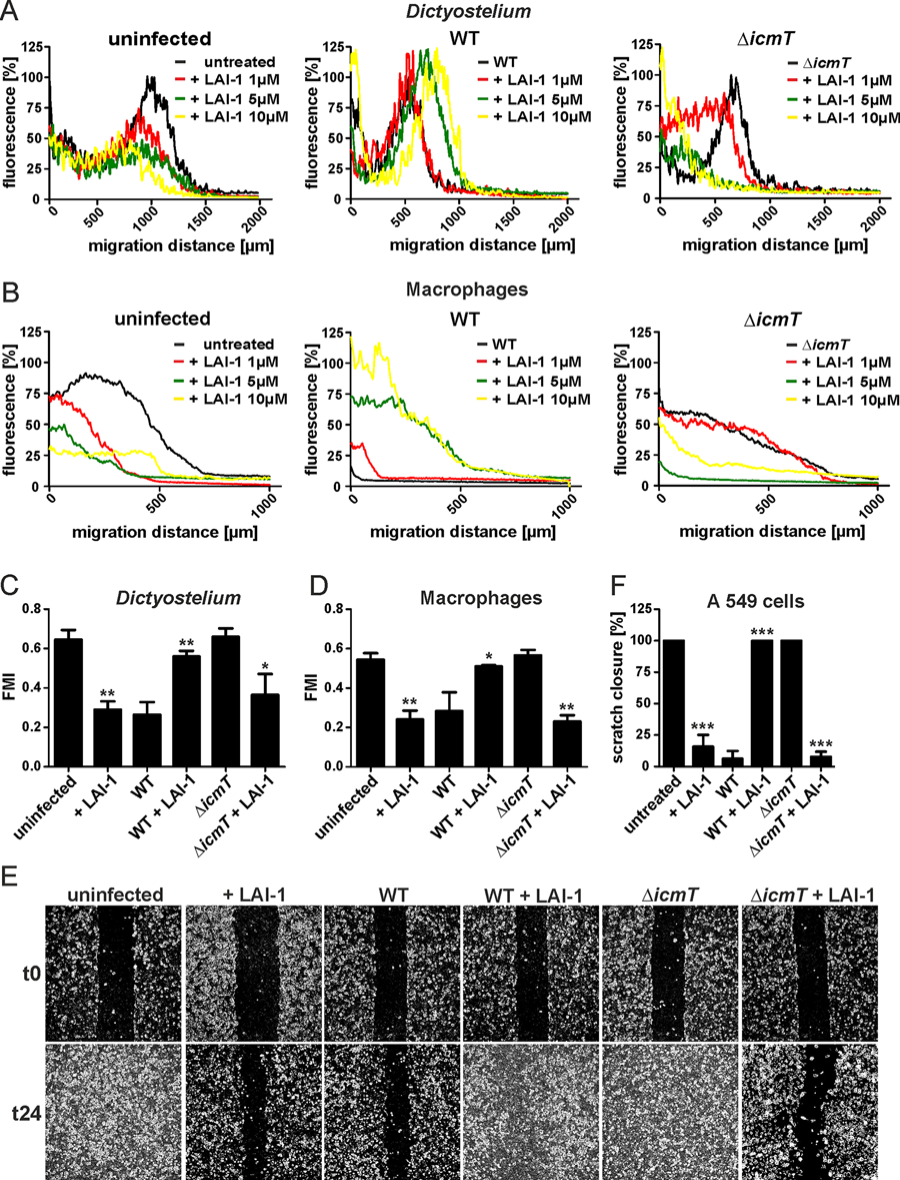
LAI-1 reverses Icm/Dot-dependent inhibition of migration by *L*. *pneumophila*. (A) *D*. *discoideum* Ax3 amoebae harboring pSW102 (GFP) or (B) RAW 264.7 macrophages were left uninfected or infected (MOI 10, 1 h) with *L*. *pneumophila* wild-type or Δ*icmT* mutant bacteria and treated with different concentrations of LAI-1 (1, 5 and 10 μM) or not. The effect of LAI-1 on migration of amoebae towards folate (1 mM) or macrophages towards CCL5 (100 ng/ml) was monitored in under-agarose assays for 4 hours. Macrophages were stained with Cell Tracker Green BODIPY. Graphs depict the per cent fluorescence intensity versus migration distance. (C) *D*. *discoideum* Ax3 amoebae harboring pSW102 (GFP) or (D) RAW 264.7 macrophages were left uninfected or infected (MOI 10, 1 h) with *L*. *pneumophila* wild-type or Δ*icmT* mutant bacteria and treated with LAI-1 (10 μM, 1 h) or not. Single cell migration towards folate (1 mM) or CCL5 (100 ng/ml) was tracked in an under-agarose assay for 15 min or 1 h, respectively. Motility parameters (forward migration index, FMI, and velocity (S7 Fig)) were analyzed using the ImageJ manual tracker and Ibidi chemotaxis software. (E) Confluent cell layers of A549 epithelial cells were left uninfected or infected (MOI 10, 1 h) with *L*. *pneumophila* wild-type or Δ*icmT* mutant bacteria, treated with LAI-1 (10 μM) or not, scratched and let migrate for 24 h. Prior to imaging (0, 24 h), the detached cells were washed off. (F) The scratch area was quantified at 7 different positions per condition using ImageJ software. Means and standard deviations of triplicate samples per condition are shown, which are representative of 3 independent experiments (C, D, F; means and standard deviations; *p < 0.05; **p < 0.01; ***p < 0.001).

*L*. *pneumophila* inhibits the migration of free-living amoebae as well as mammalian cells dependent on the Icm/Dot T4SS [25] as well as through LAI-1 (Fig 2). To test, whether synthetic LAI-1 affects Icm/Dot-dependent inhibition of cell migration, we infected *D*. *discoideum* amoebae or RAW 264.7 macrophages with wild-type *L*. *pneumophila* or Δ*icmT* mutant bacteria and treated the infected phagocytes with different concentrations of LAI-1 (Figs 7 and S7A). In under-agarose assays LAI-1 dose-dependently reversed the inhibitory effect of wild-type *L*. *pneumophila* on the migration of *D*. *discoideum* (Fig 7A) or macrophages (Fig 7B). In contrast, LAI-1 dose-dependently inhibited cell migration in phagocytes infected with Δ*icmT* mutant bacteria similar to uninfected cells.

Next, we investigated the effects of LAI-1 on the motility parameters (forward migration index and velocity) of single *D*. *discoideum* cells infected with wild-type or Δ*icmT L*. *pneumophila*. This single cell tracking analysis revealed that 10 μM LAI-1 completely reversed the inhibitory effect of wild-type *L*. *pneumophila* on the forward migration index of infected *D*. *discoideum* (Fig 7C) or macrophages (Fig 7D), but had no effect on the velocity of infected amoebae or macrophages (S7B Fig). 10 μM LAI-1 reduced the forward migration index of uninfected or Δ*icmT*-infected phagocytes to a similar extent. Taken together, LAI-1 dose-dependently reverted the effects on migration of wild-type or Δ*icmT*-infected phagocytes by affecting the forward migration index but not the velocity of the cells.

We also tested whether the inhibition of scratch wound closure by wild-type *L*. *pneumophila* is reversed by LAI-1. Confluent layers of A549 cells were infected with *L*. *pneumophila* wild-type or Δ*icmT* mutant bacteria, treated or not with 10 μM LAI-1, scratched and let migrate for 24 h (Fig 7E). As above, LAI-1 reversed the migration inhibition of wild-type bacteria, but prevented scratch closure of cells infected with Δ*icmT L*. *pneumophila* (Fig 7F). In summary, these results suggest that the exogenously added quorum sensing compound LAI-1 and intracellular *L*. *pneumophila* antagonistically target a signaling pathway to inhibit the migration of eukaryotic cells.

### Migration inhibition by *L*. *pneumophila* is augmented in absence of Cdc42

**Fig 8:**
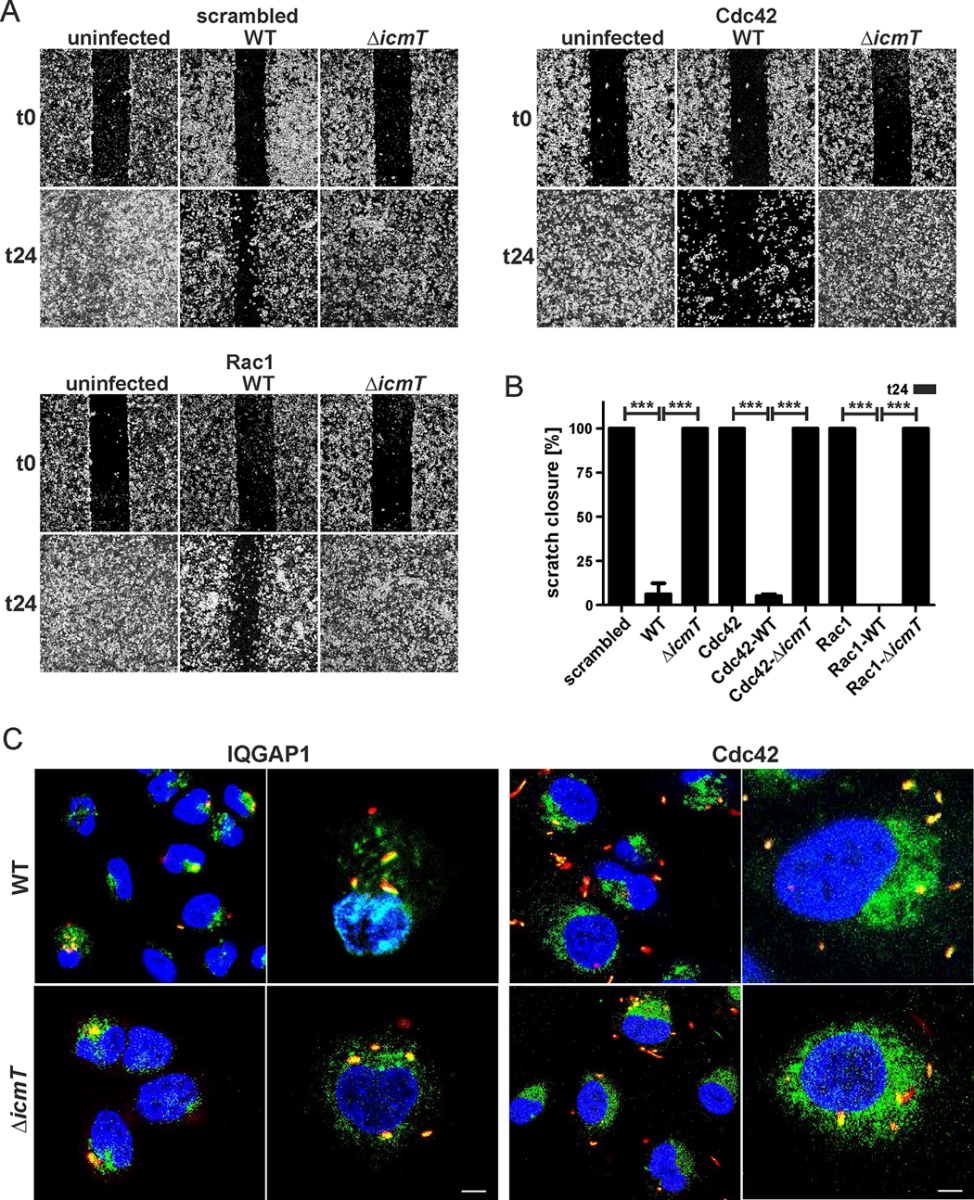
Migration inhibition by *L*. *pneumophila* is augmented in the absence of Cdc42. (A) Confluent cell layers of A549 cells were treated with (A) scrambled siRNA or siRNA against (B) Cdc42 or (C) Rac1 for 2 days, left uninfected or infected (MOI 10, 1 h) with *L*. *pneumophila* wild-type or Δ*icmT* mutant bacteria, scratched and let migrate for 24 h. Prior to imaging (0, 24 h), the detached cells were washed off. (B) The scratch area was quantified after 24 h at 7 different positions per condition using ImageJ software. Means and standard deviations of triplicate samples per condition are shown, which are representative of 3 independent experiments (***p < 0.001). (C) *L*. *pneumophila* colocalizes with IQGAP1 and Cdc42. A549 cells were infected (MOI 10, 1 h) with *L*. *pneumophila* wild-type or Δ*icmT* mutant bacteria harboring plasmid pSW001 (DsRed), and the subcellular localization of the scaffold protein (green; FITC) or the small GTPase (green; FITC) was analyzed by confocal microscopy using antibodies against IQGAP1 or Cdc42. Nuclei were stained with DAPI (blue). Bars, 5 μm.

To test the hypothesis that migration inhibition by LAI-1 or *L*. *pneumophila* converges on common host factors, we depleted the small GTPases Cdc42 or Rac1 in A549 cells prior to an infection with wild-type or Δ*icmT* mutant *L*. *pneumophila* (Fig 8A). The depletion of Cdc42 (but not Rac1) markedly further augmented the inhibition of cell migration by *L*. *pneumophila* (Fig 8B). In contrast, the depletion of Cdc42 (or Rac1) had no effect on A549 cells infected with the Δ*icmT* mutant strain. A549 cells depleted for Cdc42 and concomitantly infected with wild-type *L*. *pneumophila* appeared normal, and no increased cytotoxicity was observed.

Moreover, confocal immuno-fluorescence microscopy revealed that IQGAP1 as well as Cdc42 co-localize with *L*. *pneumophila* wild-type or Δ*icmT* mutant bacteria in infected A549 cells (Fig 8C). Yet, LAI-1 did not affect the co-localization of the bacteria with either the scaffold protein or the small GTPases (S8 Fig). Finally, neither Cdc42 nor IQGAP1 appeared to play a role for intracellular replication of *L*. *pneumophila* (S9 Fig). Collectively, these results indicate that the small GTPase Cdc42 is an essential component of the migration signal transduction pathway inhibited either by LAI-1 or by wild-type *L*. *pneumophila*, and the bacteria co-localize with Cdc42 as well as with IQGAP1 in infected cells.

## Discussion

Bacterial quorum sensing signals are not only implicated in population density-dependent signaling and gene regulation of prokaryotes, but also have an impact on eukaryotic cells in a process called inter-kingdom signaling [36]. However, little is known on a molecular level about the response of mammalian and protozoan cells to prokaryotic quorum sensing signals. The results presented here indicate that the *L*. *pneumophila* quorum sensing compound LAI-1 inhibits the directed migration of eukaryotic cells through a signaling pathway involving IQGAP1, Cdc42 and ARHGEF9. To our knowledge this is the first analysis of host factors comprising a signaling pathway implicated in inter-kingdom signaling of a bacterial AHK compound.

The compounds (*R*)-LAI-1 and also (*R*)-amino-LAI-are biologically more active as inhibitors of eukaryotic cell migration than the corresponding (*S*)-enantiomers (Fig 2B). In contrast, the (*S*)-enantiomer of the *V*. *cholerae* AHK autoinducer CAI-1 and its derivatives are more active than the corresponding (*R*)-enantiomers–at least for bacterial cell-cell communication [6, 37]. It is presently unclear, whether this observation reflects inter-kingdom versus inter-bacterial signaling. The structural determinants of CAI-1 and derivatives but not the stereochemistry of the compounds have been tested for inter-kingdom signaling responses of *Caenorhabditis elegans* [38].

Upon depletion of IQGAP1 by RNAi, treatment with LAI-1 no longer abolished the migration of A549 epithelial cells (Fig 3). IQGAP1 is a 189 kDa multi-domain scaffold protein, which harbors among others a calmodulin-binding IQ domain and a GRD Ras GAP-related domain [31, 32]. IQGAP1 is widely expressed and conserved among eukaryotes; and IQGAP-like proteins (Dgap1, GapA, RgaA) are also present in *D*. *discoideum* [39, 40] as well as on LCVs purified from the infected amoebae [30]. The scaffold protein IQGAP1 integrates multiple signaling pathways and coordinates a plethora of cellular activities, including chemokine- and growth factor-dependent cell proliferation, adhesion, migration and phagocytosis [31, 32]. To date, over 90 interacting proteins have been identified, including the small GTPases Cdc42, Rac1 and RhoA. The GRD domain does not function as a GAP, but rather binds to and stabilizes activated (GTP-bound) Cdc42 or Rac1 by inhibiting the intrinsic GTPase activity [41, 42]. Accordingly, overexpression of IQGAP1 increases the amount of active Cdc42 and Rac1 in cells, whereas depleting endogenous IQGAP1 substantially decreases the activity of both small GTPases [42].

Given the pivotal role of IQGAP1 in modulating the actin cytoskeleton through Cdc42 and Rac1, we depleted these and other small GTPases and assayed LAI-1-mediated inhibition of cell migration. The depletion of Cdc42 abrogated LAI-1-mediated cell migration inhibition, while depletion of Rac1 did not affect migration inhibition (Fig 3). Therefore, Cdc42 is clearly involved in LAI-1-mediated inter-kingdom signaling, while Rac1 is dispensable. The depletion of RhoA alone already significantly reduced the migration of A549 cells, which was not further inhibited by LAI-1. While this observation might argue for a role of RhoA in LAI-1-mediated inter-kingdom signaling, there is no evidence that IQGAP1 binds RhoA, and therefore, this small GTPase is probably not involved in the pathway. However, at this point we cannot rule out that RhoA promotes LAI-1 inter-kingdom signaling in an IQGAP1-independent way.

Treatment with LAI-1 caused the inactivation of Cdc42, i.e. lower amounts of GTP-bound Cdc42 were present in the cells (Fig 4A). Interestingly, the depletion of the Cdc42 activator ARHGEF9 abolished LAI-1-mediated cell migration inhibition, whereas the depletion of several other Cdc42 modulators had no effect (Fig 6). The Cdc42-specific GEF ARHGEF9 is up-regulated by the oncogene transcription factor CHD1L and has been previously implicated in tumor cell migration, invasion and metastasis by increasing cell motility and inducing filopodia formation [43, 44]. In the LAI-1-affected signaling pathway, ARHGEF9 seems indispensable to activate Cdc42 and promote cell migration. Collectively, these results suggest that LAI-1 (directly or indirectly) inactivates ARHGEF9 or prevents its activation and thus reduces the amount of GTP-bound Cdc42, thereby impeding cell migration.

The scaffold protein IQGAP1 and the small GTPase Cdc42 are conserved among eukaryotes; e.g. there are human and *D*. *discoideum* putative orthologues of similar size, which are overall more than 30% or 60% identical, respectively. Moreover, the proteins are also found in other amoebazoa, including *Acanthamoeba* or *Entamoeba* spp. In contrast, an ARHGEF9 homologue is apparently not present in *D*. *discoideum* or other protozoa, and therefore, in these cells the LAI-1-dependent activation of Cdc42 apparently proceeds through another GEF.

**Fig 9:**
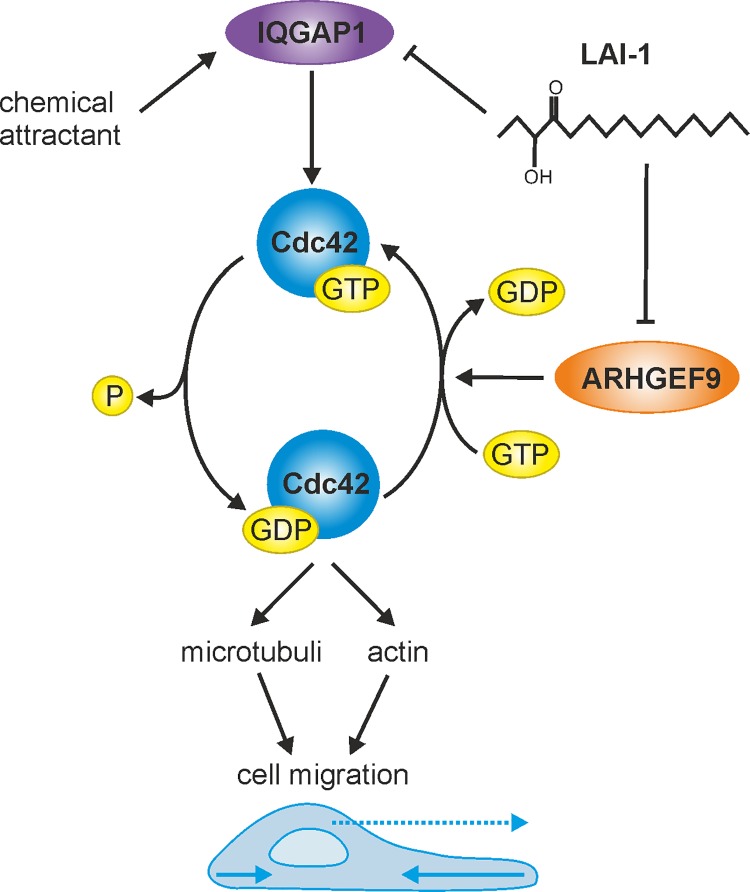
Model of LA1-1-dependent inter-kingdom signaling through IQGAP1, Cdc42 and ARHGEF9. In eukaryotic cells, LAI-1 (directly or indirectly) inhibits or prevents the activation of the Cdc42-specific GEF ARHGEF9, which in turn prevents the IQGAP1-dependent activation of Cdc42. The host cell might detect extracellular and/or intracellular LAI-1.

Upon depletion of Cdc42, IQGAP1 still redistributed to the cell cortex (Fig 5). This finding suggests that in the LAI-1-dependent signaling pathway IQGAP1 is functioning “upstream” or at the level of Cdc42. Thus, in this pathway and in the A549 cells used, IQGAP1 is a regulator of Cdc42. It is discussed quite controversially in the literature, whether IQGAP1 functions as a regulator of Cdc42 (and Rac1), an effector of the small GTPase, or both, and whether the function is specific for a given cell-type or signaling pathway [31]. Collectively, our data suggest the following mechanism of LAI-1-mediated cell migration inhibition: LAI-1 (directly or indirectly) inhibits or prevents the activation of the Cdc42-specific GEF ARHGEF9, which in turn prevents the IQGAP1-dependent activation of Cdc42 and perhaps also its stabilization by IQGAP1 (Fig 9).

The scaffold protein IQGAP1 has been implicated in the interactions of a number of intracellular pathogens or their products with host cells. While intracellular replication of *L*. *pneumophila* in A549 cells is not affected by the depletion of IQGAP1 (S9 Fig), invasion by *Salmonella enterica* Typhimurium (*S*. *typhimurium*) of embryonic fibroblasts from mice lacking IQGAP1 is decreased, and Cdc42 as well as Rac1 activation is abrogated [45]. *S*. *typhimurium* also subverts host cell motility and migration. The vacuolar pathogen promotes chronic infection by translocating through the SPI2 type III secretion system (T3SS) the effector SseI (*alias* SrfH), which directly binds to IQGAP1 and inhibits the motility of immune phagocytes such as primary macrophages and dendritic cells [46, 47]. Analogously, *L*. *pneumophila* abolishes cell migration in a manner dependent on the Icm/Dot T4SS. However, whereas the effector LegG1, a Ran activator, was found to antagonize Icm/Dot-dependent cell migration inhibition, an effector inhibiting cell migration has not been identified yet [25]. In any case, *L*. *pneumophila* abolishes cell migration likely by an Icm/Dot-translocated effector protein as well as by the low molecular weight signaling molecule LAI-1, and migration inhibition triggered by *L*. *pneumophila* or LAI-1 proceeds through a common host factor, Cdc42 (Fig 8). IQGAP1 is also involved in modulation of cell migration by obligate intracellular bacteria of the genus *Chlamydia*. *Chlamydia pneumoniae* promotes cell adhesion and migration of vascular smooth muscle cells through IQGAP1 [48], while *Chlamydia trachomatis* impairs the migration of HeLa epithelial cells [49] and endosymbiotic environmental *Chlamydia* spp. control the motility of their host, *Acanthamoeba* sp. [50].

Finally, the AHL autoinducer *N*-3-oxo-dodecanoyl-L-homoserine lactone from *P*. *aeruginosa* has been implicated in migration inhibition of epithelial cells through an IQGAP1-dependent pathway. The AHL quorum sensing molecule alters cell migration by apparently interacting with IQGAP1, which upon phosphorylation of the small GTPases Rac1 and Cdc42 alters its cellular localization [51]. Yet, in this study the autoinducer was effective only at very high concentrations (200 μM) and further factors comprising the signaling pathway have not been identified.

It is currently unknown under what physiological conditions and at which concentrations LAI-1 is produced by *L*. *pneumophila* or how the compound is secreted. Yet, the findings that overproduction of *lqsA* complements the defect in cell inhibition of an Δ*lqsA* mutant strain and leads to inhibition of cell migration by a Δ*icmT* mutant suggest that under these conditions LqsA (and in consequence, LAI-1) are produced. Possibly, *L*. *pneumophila* also produces LAI-1 in biofilms. In this setting, the adherent cells could secrete the signaling compound, thus inhibiting protozoa migration and increasing the likelihood that the pathogen is taken up by a potential host cell. At this point, we do not have any evidence suggesting that LAI-1 increases infectivity. Rather, treatment of *D*. *discoideum* or RAW 264.7 macrophages with up to 10 μM LAI-1 did not affect the uptake of wild-type or Δ*icmT* mutant *L*. *pneumophila* (S1 Fig). In summary, our study reveals that the *L*. *pneumophila* autoinducer LAI-1 inhibits the migration of eukaryotic cells in the low micromolar range through a signaling pathway involving the host factors IQGAP1, Cdc42 and ARHGEF9. These findings provide the basis for a further mechanistic analysis of how *L*. *pneumophila* impedes cell migration and benefits from this strategy.

## Materials and Methods

### Synthesis of LAI-1 and amino-LAI-1

**Fig 10:**
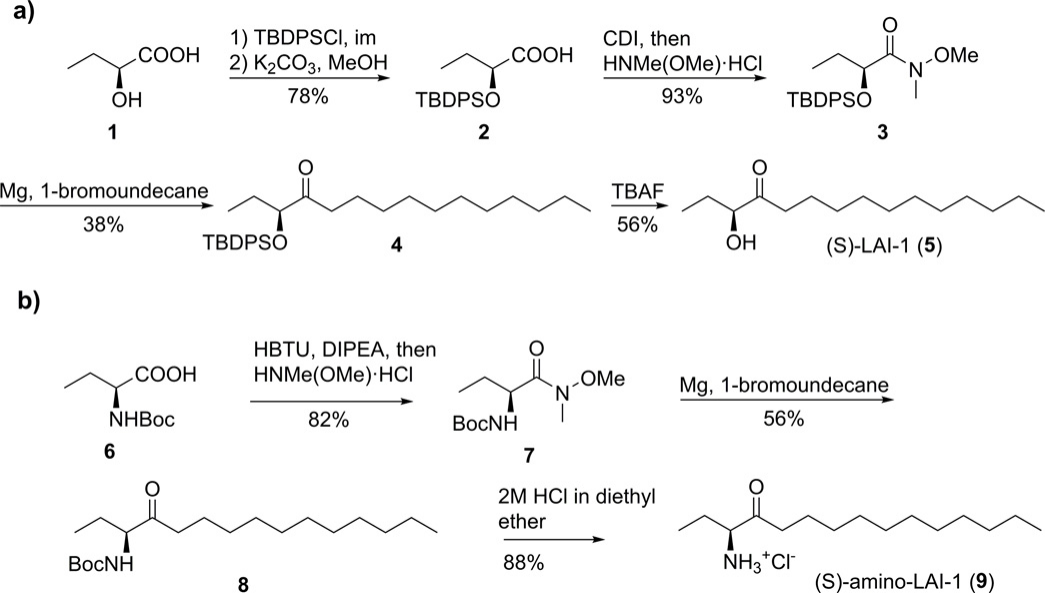
Chemical synthesis of LAI-1 and amino-LAI-1. The chemical synthesis of the putative *L*. *pneumophila* small molecule compounds (A) LAI-1 and (B) amino-LAI-1 is shown.

The chemical synthesis of (*S*)-LAI-1 (**5**) (Fig 10) started with commercially available (*S*)-2-hydroxybutyric acid **1** that was protected as a TBDPS ether by a two-step protocol to yield carboxylic acid **2**, analogous to the synthesis of 3-hydroxytridecane-4-one (CAI-1) [6]. Formation of the Weinreb amide **3** followed by reaction with undecanemagnesium bromide provided ketone **4**. Final deprotection with TBAF and HPLC purification gave LAI-1 (**5**) in pure form (Fig 10A). (*R*)-LAI-1 was synthesized analogously starting from (*R*)-2-hydroxybutyric acid.

Enantiopure (*S*)-amino-LAI-1 (**9**) as its HCl-salt was synthesized as follows. *(S)-*N-Boc-Abu **6** was converted into the corresponding Weinreb amide **7** via HBTU activation, which was treated with undecanemagnesium bromide to provide the corresponding ketone **8** after silica gel chromatography. Next, the N-Boc-ketone derivative **8** was dissolved in anhydrous diethyl ether and deprotected via the treatment with 2 M HCl in diethyl ether. After evaporation to dryness, the resulting residue was recrystallized from anhydrous chloroform to give enantiopure crystalline *(S)-*amino-LAI-1 (**9**) as its HCl salt (Fig 10B). (*R*)-amino-LAI-1 was synthesized analogously starting from (*R*)-N-Boc-Abu carboxylic acid. LAI-1 and its derivatives were dissolved in 100% dimethylsulfoxide (DMSO) as a stock solution for the experiments. For details of the chemical syntheses see section Supporting Information.

### Bacteria, cells, growth conditions and infection

*L*. *pneumophila* strains (Table 1) were grown for 3 days on CYE agar plates [52] containing charcoal yeast extract, buffered with N-(2-acetamido)-2-amino-ethanesulfonic acid (ACES). Liquid cultures were inoculated in AYE medium—supplemented with chloramphenicol (Cm 5μg ml^-1^), if necessary—at an OD_600_ of 0.1 and grown at 37°C to an OD_600_ of 3.0 (21–22 h). *D*. *discoideum* strains were grown as described [53]. Murine RAW 264.7 macrophages and A549 lung epithelial carcinoma cells were cultivated in RPMI 1640 medium amended with 10% heat-inactivated fetal bovine serum and 1% glutamine (all from Life Technologies).

**Table 1:** Bacterial strains and plasmids used in this study.

Strain or plasmid	Relevant properties ^a^	Reference
*L*. *pneumophila*		
AK01 (Δ*lqsT*)	JR32 *lqsT*::Km	[12]
AK02 (Δ*lqsS*-Δ*lqsT*)	JR32 *lqsS*::Km *lqsT*::Gm	[12]
JR32	*L*. *pneumophila* serogroup 1 Philadelphia, salt-sensitive isolate of AM511	[62]
GS3011 (Δ*icmT*)	JR32 *icmT*3011::Km	[63]
NT02 (Δ*lqsA*)	JR32 *lqsA*::Km	[16]
NT03 (Δ*lqsR*)	JR32 *lqsR*::Km	[10]
NT05 (Δ*lqsS*)	JR32 *lqsS*::Km	[16]
Plasmids		
pSW001	pMMB207-C-*DsRed*, Δ*lacI* ^q^, *dsred* (constitutive)	[64]
pNT28	pMMB207C, *gfp* (constitutive)	[10]
pNT36	pMMB207C, *gfp* (constitutive), *lqsA* (P*lqsA*)	[16]

^a^ Abbreviations: Km, kanamycin resistance; Gm, gentamicin resistance.

The infection of phagocytes with *L*. *pneumophila* was analyzed as described using *D*. *discoideum*, murine RAW 264.7 macrophages or A549 cells [10, 53–56]. Briefly, cells were infected (MOI 10) with *L*. *pneumophila* grown for 21–22 hours in AYE broth, the infection was synchronized by centrifugation [450 × *g*, 10 min, room temperature (RT)], and the infected phagocytes were incubated at 23°C (*D*. *discoideum*) or at 37°C/5% CO_2_ (mammalian cells) for the indicated time.

### Under-agarose assay and single cell tracking

Under-agarose assays using *D*. *discoideum* Ax3 pSW102 (GFP) were performed as described [25, 57]. Briefly, microscopy dishes (μ-Dish, 35 mm, Ibidi) were filled with a mixture of melted agarose in SM medium [10 g bacteriological peptone (Oxoid), 1 g Bacto yeast extract (BD Biosciences), 1.9 g KH_2_PO_4_, 0.6 g K_2_HPO_4_, 0.43 g MgSO_4_, 10 g glucose per liter, pH 6.5]. After solidification, 3 parallel slots of 2 × 4 mm (for cells and chemo-attractant solution) were cut 5 mm apart into the agarose (Fig 1A). The chemo-attractant solution, 1 mM folic acid (Sigma-Aldrich) in SM medium, was filled into the central slot 30 min before the cell suspensions were filled into the neighboring slots. Prior to the experiments, 10^6^ *D*. *discoideum* Ax3 pSW102 (GFP) cells were seeded onto 6-well plates overnight in HL-5 medium. The amoebae were washed once with MB medium [14 g bacteriological peptone (Oxoid), 7 g Bacto yeast extract (BD Biosciences), 4.26 g MES (Sigma-Aldrich) per liter, pH 6.9] and kept for 1 h in 3 ml MB medium. During this period LAI-1, CAI-1 or derivatives were added at the concentrations indicated and DMSO was used as a negative control. If indicated, infections with *L*. *pneumophila* were performed in parallel at an MOI of 10, for 1 h at 23°C. After 2 washing steps with MB medium (10 min centrifugation, 1500 rpm), the amoebae were detached by scratching into 500 μl MB, and 30 μl of the cell suspension was filled into the slots. The dishes were incubated in a humid chamber and the cells let migrate for 4 h at 23°C.

Under-agarose assays using murine RAW 264.7 macrophages were performed as described [25, 58]. The microscopy dishes (μ-Dish, 35 mm; Ibidi) were incubated with 10% FCS, for 30 min at RT. After washing twice with PBS, the dish was filled with 1% UltraPure agarose in a 1:1 mixture of RPMI/HBSS (Life Technologies). Three parallel slots (5 mm part) were formed using a template, and the chemo-attractant solution (CCL5, 100 ng ml^-1^, Invitrogen) was placed in the middle for 1 h min, before the cell suspensions were filled into the neighboring slots. Prior to the experiments 10^6^ macrophages were seeded onto 6-well plates in RPMI and incubated overnight at 37°C. LAI-1, CAI-1 or derivatives were added at the concentrations indicated for 1 h. If indicated, infections with *L*. *pneumophila* were performed in parallel at an MOI of 10, for 1 h at 37°C. After two washing steps with RPMI, the cells were incubated for 45 min with 1 μM CellTracker BODIPY (green), washed again twice and kept in 3 ml RPMI. The cells were detached by scratching into 500 μl RPMI, 150 μl was placed into the slots and let migrate for 4 h in a humid chamber at 37°C.

Under-agarose cell migration of macrophages (labelled with BODIPY) or *D*. *discoideum* (producing GFP) was analyzed by fluorescence microscopy using a Leica TCS SP5 confocal microscope (HCX PL APO CS 10×/0.40 dry UV objective, Leica Microsystems). The tile scan function of the Leica software allowed obtaining merged overview pictures. Cell migration was quantified using ImageJ software (version 1.45, function ‘plot profile’). The fluorescence intensities of infected cells relative to uninfected cells were plotted against the migration distance. 100% fluorescence is defined as the maximum fluorescence intensity of the untreated control sample, i.e. uninfected cells or cells infected with wild-type or Δ*icmT* mutant *L*. *pneumophila*.

Individual phagocytes (*D*. *discoideum* or macrophages) were tracked in the under-agarose assay using a SP5 confocal microscope (HCX PL APO CS 40×/1.25 oil UV objective) essentially and the ImageJ manual tracking plugin (‘chemotaxis and migration tool 2.0’, Ibidi) as described [25]. *D*. *discoideum* cells were filled into the slots, and monitored after 1 h for 15 min at 23°C within a 2 h time window by taking 1 frame per 25 s. RAW 264.7 macrophages were tracked at 2 h post infection at 37°C during another 1 h with 1 frame per 35 s.

### *In vitro* scratch assay

*In vitro* scratch assays were performed as described [25, 49, 59]. Briefly, A549 cells were seeded into 35 mm μ-Dishes (Ibidi) at a density of 1.5 × 10^5^ cells ml^−1^ (3 × 10^5^ cells/dish) and incubated for 24 h. Confluent cell layers were washed with fresh medium and infected for 1.5 h with *L*. *pneumophila* strains (MOI 10) and/or treated with 10 μM LAI-1. After the infection and/or compound treatment, the cell layer was scratched with a sterile pipette tip and washed with fresh medium to remove detached cells. Images of the scratched positions were taken at time point zero and after 24 h using a Leica SP5 confocal microscope (HCX PL APO CS 10×/ 0.40 dry UV objective). The percentage of ‘scratch closure’ was quantified using ImageJ software (function ‘analyze particles’) by comparing the remaining scratch area with the initial cell-free scratch area (S2 Fig).

### RNA interference

For RNAi experiments in scratch assays, A549 cells were grown in 35 mm μ-Dishes (Ibidi) and treated for 2 d with a final concentration of 10 nM of a mixture of 4 different siRNA oligonucleotides (S3 Table). To this end, the siRNA stock (10 μM) was diluted 1:15 in RNAse-free water, and 22.5 μl of diluted siRNA was added per well. Allstars siRNA (Qiagen) was used as a negative control. Subsequently, 181.9 μl RPMI medium without FCS was mixed with 5.6 μl HiPerFect transfection reagent (Qiagen), added to the well, mixed and incubated for 5–10 min at RT. In the meantime, cells were diluted in RPMI medium with 10% FCS, 1.312 ml of the diluted cells (1.5 × 10^5^ cells ml^-1^) were added on top of each siRNA-HiPerFect transfection complex and incubated for 48 h. After a washing step with fresh RPMI medium, cells were infected with *L*. *pneumophila* strains and/or treated with 10 μM LAI-1, and the scratch assay was performed as described above. The depletion efficiency of all siRNA oligonucleotides was assessed by Western blot (S3 Fig) using antibodies against IQGAP1, Cdc42, RhoA, Rac1, ARHGEF9, ARHGAP17, DOCK11, FGD1, GAP1, CD2AP or GAPDH (Abcam, 1:1,000).

For RNAi experiments in growth assays, A549 cells were grown in 96-well plates and treated for 2 d with a final concentration of 10 nM of the siRNA oligonucleotides indicated (S3 Table). To this end, the siRNA stock (10 μM) was diluted 1:15 in RNAse-free water, and 3 μl of diluted siRNA was added per well. Allstars siRNA (Qiagen) was used as a negative control. Subsequently, 24.25 μl RPMI medium without FCS was mixed with 0.75 μl HiPerFect transfection reagent (Qiagen), added to the well, mixed and incubated for 5–10 min at RT. In the meantime, cells were diluted in RPMI medium with 10% FCS, 175 μl of the diluted cells (2 × 10^4^ cells) were added on top of each siRNA-HiPerFect transfection complex and incubated for 48 h. The cells were then infected (MOI 10) with GFP-producing *L*. *pneumophila* grown for 21 h, diluted in RPMI, centrifuged and incubated for 1 h. The infected cells were washed 3 times with pre-warmed medium containing 10% FCS and incubated for 24 h (the plate was kept moist with water in extra wells). Intracellular bacterial growth was measured by fluorescence using a plate reader (FluoStar Optima, BMG Labtech).

### Treatment of *D*. *discoideum* with LAI-1, microarray analysis and real time PCR

Exponentially growing *D*. *discoideum* Ax2 wild-type cells (1–4 × 10^6^ cells/ml) were either treated with 20 μM of synthetic LAI-1 in DMSO or mock-treated with DMSO only. After 3 h 1 × 10^7^ cells were harvested, washed twice with Soerensen phosphate buffer (2 mM Na_2_HPO_4_, 15 mM KH_2_PO_4_, pH 6.0), and total RNA was isolated (Qiagen RNeasy mini kit) using the protocol for isolation of cytoplasmic RNA. In total six microarrays with dye swaps for each isolation were analyzed with labelled cDNAs derived from three independent RNA isolations. Microarray analysis was essentially carried out as described [33, 60]. Oligonucleotide primers for quantitative RT-PCR (S4 Table) were designed on the basis of sequence information, selected with the Primer 3 program (http://bioinfo.ut.ee/primer3-0.4.0/primer3/) and purchased from Metabion Corp. (Munich, Germany). Reverse transcription and RT-PCR were essentially performed as described [33].

### Pulldown and western blot

Cdc42(GTP) and Cdc42(GDP) were identified in epithelial cells by pulldown experiments, followed by Western blot. To this end, A549 cells treated or not with 10 μM LAI-1 were suspended in ice cold RIPA buffer and incubated at 4°C for 10 min. Cellular debris was pelleted by centrifugation (10 min, 10,000 × *g*, 4°C). 1 mL of the supernatant (100–500 μg total cell protein) was incubated for 1 h at 4°C with glutathione resin and GST-PBD_Pak1_ (the p21-binding domain (PBD) of p21-activated protein kinase (PAK1) specifically binds to active Cdc42) according to the manufacturer’s recommendation (Thermo Scientific). As controls, cell lysates were treated with GTPγS or GDP to yield the active or inactive form of Cdc42. Subsequent Western blot was performed with an antibody recognizing Cdc42(GTP/GDP) (Abcam, 1:1,000).

Alternatively, the supernatant was transferred to a fresh centrifuge tube on ice, together with 20 μl of resuspended protein A/G PLUS agarose slurry (Santa Cruz), incubated at 4°C for 30 min and pelleted by centrifugation (2,000 × *g*, 5 min, 4°C). The lysate was then incubated with primary anti-Cdc42(GTP/GDP) antibody (Abcam, 1:1,000), 20 μl of resuspended AG PLUS agarose was added and incubated on a rotating device for 1 h at 4°C. Immuno-precipitates were collected by centrifugation (2,000 × *g*) for 5 min at 4°C. The pellet was washed 4 times with 1 ml of RIPA buffer and after the final wash step resuspended in 40 μl of loading buffer. After boiling for 2–3 min, samples were subjected to SDS-PAGE and analyzed by Western blot using anti-Cdc42(GTP/GDP) or anti-Cdc42(GTP) antibodies (Abcam, 1:1,000). The amount of GAPDH, actin or α-tubulin in cell lysates was determined by Western blot using polyclonal antibodies (Abcam, 1:1,000–1:2000).

### Uptake and cytotoxicity assays

For uptake experiments, *D*. *discoideum* (5 × 10^5^) or RAW 264.7 macrophages (2.5 × 10^5^) were infected (MOI 10) for 1 h with GFP producing *L*. *pneumophila* wild-type or ∆*icmT* mutant bacteria and treated with different concentrations of LAI-1 (1, 5 or 10 μM). Fluorescence of GFP-positive phagocytes was determined by flow cytometry [61].

To determine cytotoxicity of *L*. *pneumophila* strains or LAI-1, *D*. *discoideum* or macrophages were seeded into 24 well plates, infected with *L*. *pneumophila* (MOI 10, 4 h) and collected by scraping into 15 ml tubes. After centrifugation (240 × *g*, 10 min), the cells were resuspended in 500 μl SorC (*D*. *discoideum*) or PBS (macrophages). Propidium iodide (PI) solution (2.5 μg μl^-1^) was added to the tubes, incubated for 10 min in the dark, and the PI-positive cells were analyzed by flow cytometry [61].

### Immuno-fluorescence microscopy

For immuno-fluorescence analysis, A549 cells were seeded on coverslips in a 24 well plate, treated or not with LAI-1 (10 μM) and infected or not with *L*. *pneumophila* wild-type or Δ*icmT* strains. Cells were fixed with 3% paraformaldehyde for 15 min, washed three times with PBS, permeabilized with 0.1% Triton X-100 and blocked with 1% BSA. Cells were then incubated with antibodies against IQGAP1, Cdc42(GTP/GDP) or Cdc42-phospho-Ser71 (Abcam; each 1:200). The protein amount per cell was quantified using Image J software (function “invert LUT” and “analyse/cell counter”).

The microtubule network was analyzed with RAW 264.7 macrophages, infected or not with *L*. *pneumophila* (MOI 10, 1 h). After a washing step with BRB80 (80 mM PIPES, pH 6.8, 1 mM MgCl_2_, 1 mM EGTA) the cells were fixed (50% BRB80, 0.1% Triton X-100, 0.5% glutaraldehyde) for 5 min. Subsequently, the cells were washed with SorC (Soerensen phosphate buffer containing 50 mM CaCl_2_) and blocked with 1 mg/ml sodium borohydrate in SorC for 10 min. The samples were stained with the anti-α-tubulin antibody WA3 (gift from M. Schleicher). Appropriate secondary antibodies (1:200) were used. The number of microtubule fibers was counted along cross sections in the cell. Typically, four sections per cell were considered, and each peak represents one fiber (S2 Fig). Actin was visualized in RAW 264.7 macrophages, seeded on coverslips in a 24 well-plate, using Texas red-phalloidin staining, followed by wash steps with PBS, permeabilization with cold 1% Triton X-100/PBS for 3–5 min and blocking with 1% BSA. Nuclei were stained with DAPI (0.1 μg/ml). The degree of actin re-localization was evaluation by visual inspection of single cells. If present, a layer of cortical actin was obviously visible, allowing scoring cells with or without cortical actin. The samples were analyzed with a Leica SP5 confocal microscope.

### Data analysis

Images were evaluated with ImageJ software. Further analysis was performed by using normalized background-subtracted band intensity values, defined as RIU (Relative Intensity Units). All experiments were carried out in triplicates and significance was determined using an unpaired, two-tailed Student’s *t* test.

## Supporting Information

S1 Fig Effect of LAI-1 on *L*. *pneumophila* uptake or cytotoxicity. (A) *D*. *discoideum* or (B) RAW 264.7 macrophages were left uninfected or infected (MOI 10, 1 h) with *L*. *pneumophila* wild-type or Δ*icmT* harboring pCR76 (GFP) and treated with LAI-1 (1, 5 or 10 μM, 1 h). DMSO treatment was used as control. Uptake efficiency was determined by flow cytometry (GFP-positive phagocytes). (C) *D*. *discoideum* or (D) RAW 264.7 macrophages were left uninfected or infected with *L*. *pneumophila* wild-type or Δ*icmT* (MOI 10) and treated with LAI-1 (1, 5 or 10 μM) for 4 h. DMSO treatment was used as control. Subsequently, the cells were stained with propidium iodide (PI; 2.5 μg/ml), and cytotoxicity was determined by flow cytometry.
(TIF)

S2 Fig Quantification of microtubules and scratch wound closure. (A) RAW 264.7 macrophages treated with LAI-1 (10 μM, 1 h) or not were immuno-labeled for α-tubulin (green), and microtubule fibers per cell were counted along cross-sections (left panel, yellow lines). The 4 graphs (right panel) depict the relative fluorescence intensity (arbitrary units, AU) along the 4 cross-sections of the image. Bar, 5 μm. (B) A confluent layer of A549 cells was scratched with a sterile pipette, non-adherent cells were washed away (bright field micrograph, left panel), and the scratch are was quantified using Image J software (right panel).
(TIF)

S3 Fig Analysis of siRNA depletion efficiency by Western blots. The efficiency of siRNA depletion (mixture of 4 different oligonucleotides) was assessed by Western blot using (A) antibodies corresponding to the targets indicated or (B) antibodies against Cdc42, Rac1 or IQGAP1 corresponding to possible off-targets of ARHGEF9-directed siRNA.
(TIF)

S4 Fig LAI-1-dependent inhibition of cell migration does not require Ran or CD2AP. Confluent cell layers of A549 cells were left untreated or treated for 2 days with siRNA against (A) the small GTPase Ran or its effector RanBP1, or (B, C) the SH3-domain scaffold protein CDAP2, incubated with LAI-1 (10 μM, 1h) or not, scratched and let migrate for 24 h. Detached cells were washed off prior to imaging (0, 24 h). (A, C) The scratch area was quantified after 24 h using ImageJ software. Means and standard deviations of 3 independent experiments are shown (*** *p* < 0.001). The depletion efficiency of the siRNAs was assayed by Western blot (S3 Fig, [26]).
(TIF)

S5 Fig LAI-1 promotes inactivation but does not alter phosphorylation of Cdc42. A549 cells were treated with LAI-1 (10 μM, 1 h) or not, and (A, B, D) lysed or (C) fixed. (A) Pull down with an antibody specifically recognizing Cdc42(GTP) and protein A/G agarose. The amount of active Cdc42 was analyzed by Western blot using an antibody recognizing Cdc42(GTP/GDP) (left panel). Quantification by densitometry was performed using ImageJ (right panel). Using an antibody against Cdc42/Rac1-phospho-Ser71 (B) Western blot or (C) immuno-fluorescence was performed (left panels: green, FITC; blue, DAPI; right panel: graph depicts the relative fluorescence intensity (arbitrary units, AU) along a section of a cell). Bar, 5 μm. (D) Western blots using antibodies against Cdc42, Rac1 or IQGAP1.
(TIF)

S6 Fig LAI-1-mediated gene regulation in *D*. *discoideum*. (A) Pie diagram of functionally categorised *D*. *discoideum* genes up- or down-regulated by 20 μM LAI-1. This concentration of LAI-1 led to robust changes in gene regulation, without being toxic to the amoebae. Shown are the absolute numbers of genes in different categories according to the yeast classification scheme and adapted to *D*. *discoideum*. Red and blue values in brackets indicate the percentage of up- and down-regulated genes in each category, respectively. (B) Validation of LAI-1-mediated differential expression of selected *D*. *discoideum* genes by quantitative real time (RT)-PCR using the oligonucleotides listed in S4 Table. The data indicate fold change in amoebae treated with 10 μM LAI-1 compared to control cells treated with DMSO only. Means and standard deviations of nine measurements from three independent RT-PCR experiments are shown. Red: up-regulated genes; blue: down-regulated genes.
(TIF)

S7 Fig LAI-1 reverses Icm/Dot-dependent inhibition of migration by *L*. *pneumophila*. (A) *D*. *discoideum* Ax3 amoebae harboring pSW102 (GFP) or (C) RAW 264.7 macrophages were infected (MOI 10, 1 h) with *L*. *pneumophila* wild-type or Δ*icmT* mutant bacteria and treated with LAI-1 (10 μM, 1 h) or not. Single cell migration towards folate (1 mM) or CCL5 (100 ng/ml) was tracked in an under-agarose assay for 15 min or 1 h, respectively. (B, C) Motility parameters (velocity and forward migration index, FMI (Fig 7C)) were analyzed using the ImageJ manual tracker and Ibidi chemotaxis software.
(TIF)

S8 Fig LAI-1 does not affect co-localization of *L*. *pneumophila* with Cdc42 or IQGAP1. A549 cells were infected (MOI 10, 1 h) with *L*. *pneumophila* wild-type or Δ*icmT* mutant bacteria harboring plasmid pSW001 (DsRed) and treated with LAI-1 (10 μM, 1 h), fixed and stained with antibodies against IQGAP1 or Cdc42 (green). The cellular localization of IQGAP1 or Cdc42 was analyzed by confocal fluorescence microscopy (green, FITC; blue, DAPI). Bar: 5μm.
(TIF)

S9 Fig Depletion of Cdc42 or IQGAP1 does not affect intracellular replication of *L*. *pneumophila*. A549 cells were treated with a mixture of 4 different siRNAs against either Cdc42 or IQGAP1 for 2 days and infected with *L*. *pneumophila* wild-type or Δ*icmT* mutant bacteria harboring pCR76 (GFP). Fluorescence was measured at different timepoints post-infection (1, 20, 24 and 48 h). Depletion of Cdc42 or IQGAP1 does neither affect intracellular replication of wild-type *L*. *pneumophila* nor lack of replication of Δ*icmT* mutant bacteria.
(TIF)

S1 Table Selected *D*. *discoideum* genes differentially regulated by LAI-1. (DOCX)

S2 Table All *D*. *discoideum* genes differentially regulated by LAI-1. (DOCX)

S3 Table Oligonucleotides used for RNA interference. (DOCX)

S4 Table Primer pairs used for quantitative real-time PCR analysis. (DOCX)

S1 Text Synthesis of LAI-1 and amino-LAI-1. (DOCX)
