# Supplementary figures and images for: Inter-kingdom Signaling by the Legionella Quorum Sensing Molecule LAI-1 Modulates Cell Migration through an IQGAP1-Cdc42-ARHGEF9-Dependent Pathway
Source: PLoS Pathog. 2015 Dec 3;11(12):e1005307. doi: 10.1371/journal.ppat.1005307 (PMC4669118; doi:10.1371/journal.ppat.1005307)

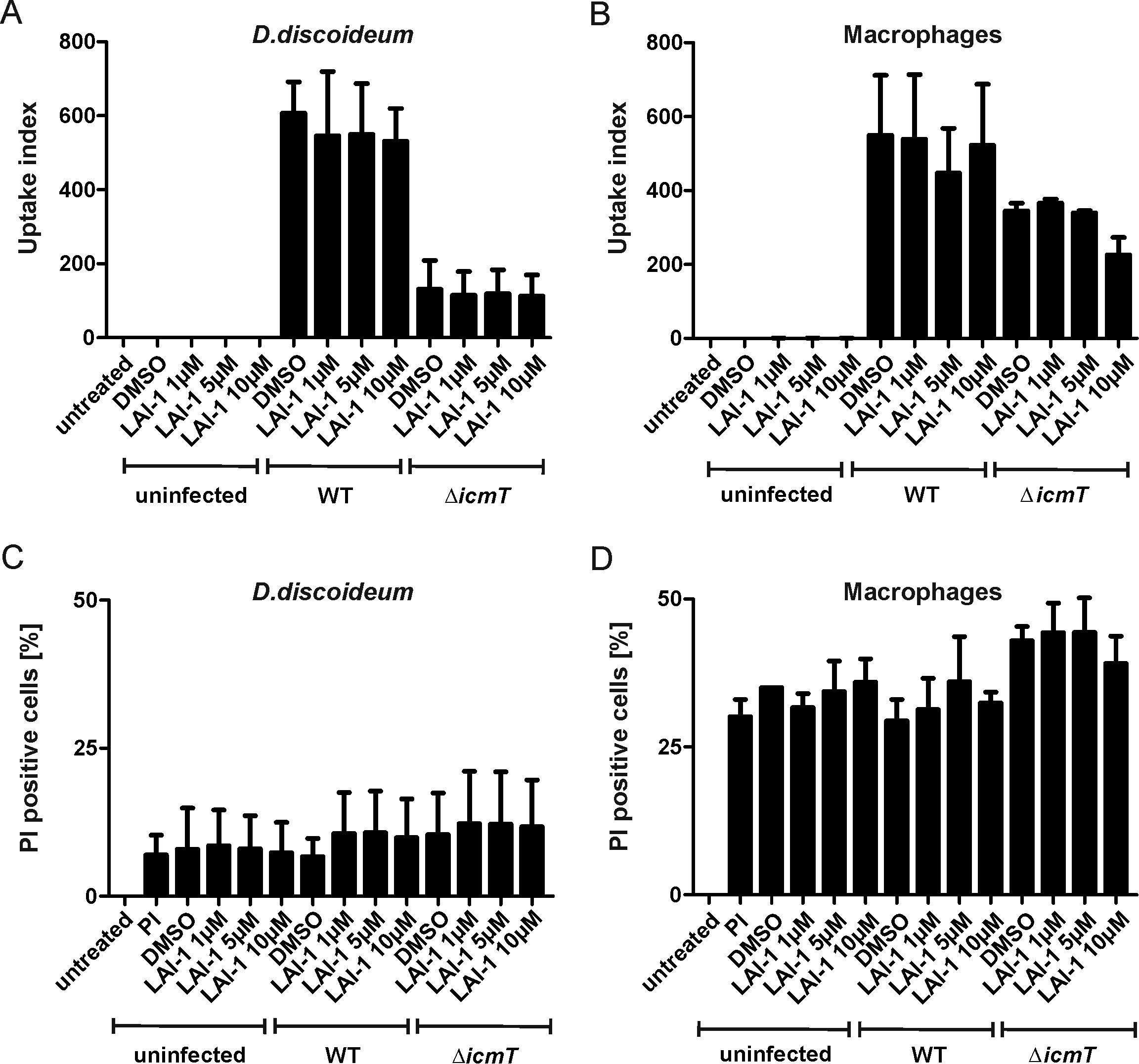

Supplement: S1 Fig — (A) D. discoideum or (B) RAW 264.7 macrophages were left uninfected or infected (MOI 10, 1 h) with L. pneumophila wild-type or ΔicmT harboring pCR76 (GFP) and treated with LAI-1 (1, 5 or 10 μM, 1 h). DMSO treatment was used as control. Uptake efficiency was determined by flow cytometry (GFP-positive phagocytes). (C) D. discoideum or (D) RAW 264.7 macrophages were left uninfected or infected with L. pneumophila wild-type or ΔicmT (MOI 10) and treated with LAI-1 (1, 5 or 10 μM) for 4 h. DMSO treatment was used as control. Subsequently, the cells were stained with propidium iodide (PI; 2.5 μg/ml), and cytotoxicity was determined by flow cytometry. (TIF) [file ppat.1005307.s001.tif]

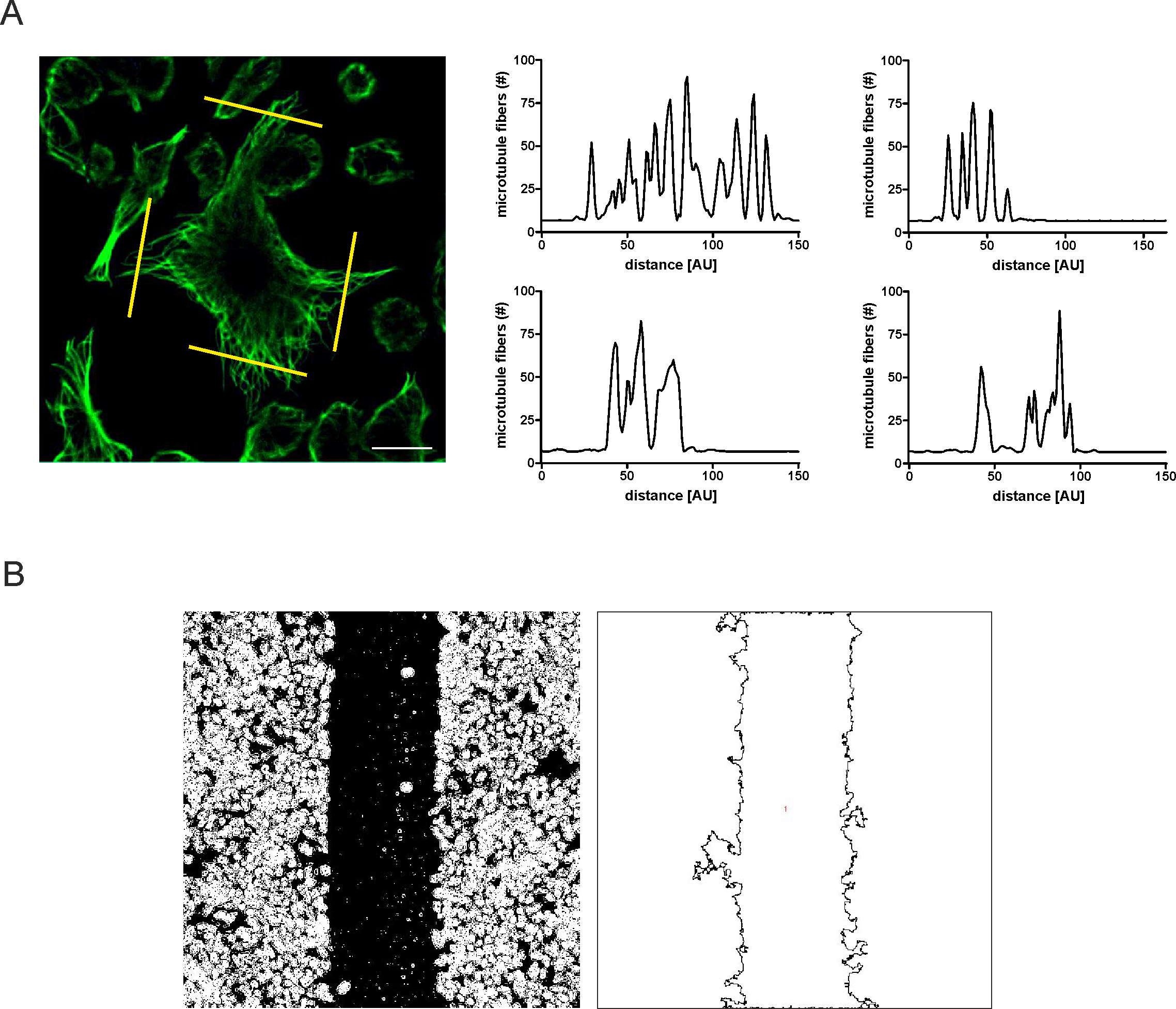

Supplement: S2 Fig — (A) RAW 264.7 macrophages treated with LAI-1 (10 μM, 1 h) or not were immuno-labeled for α-tubulin (green), and microtubule fibers per cell were counted along cross-sections (left panel, yellow lines). The 4 graphs (right panel) depict the relative fluorescence intensity (arbitrary units, AU) along the 4 cross-sections of the image. Bar, 5 μm. (B) A confluent layer of A549 cells was scratched with a sterile pipette, non-adherent cells were washed away (bright field micrograph, left panel), and the scratch are was quantified using Image J software (right panel). (TIF) [file ppat.1005307.s002.tif]

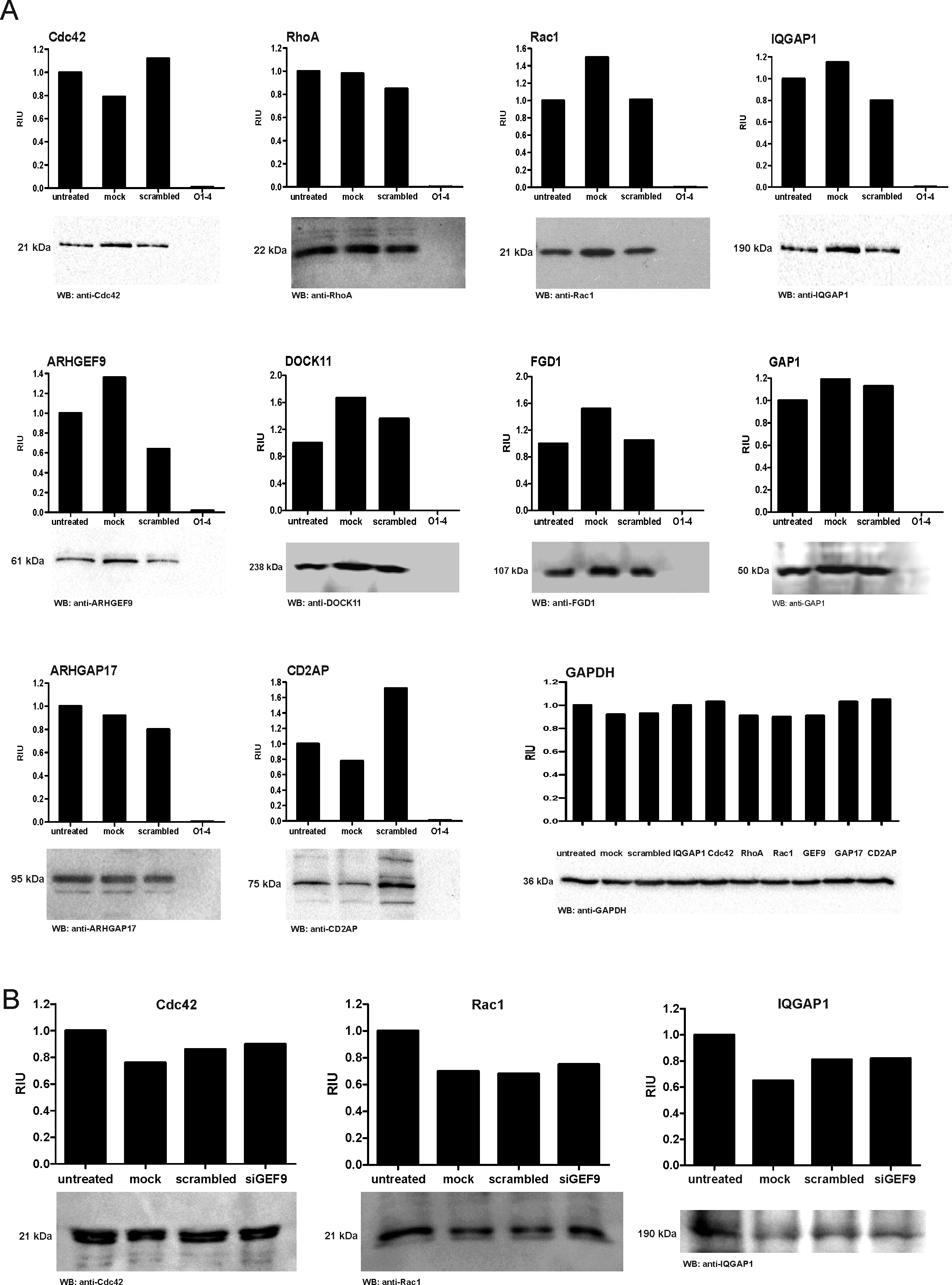

Supplement: S3 Fig — The efficiency of siRNA depletion (mixture of 4 different oligonucleotides) was assessed by Western blot using (A) antibodies corresponding to the targets indicated or (B) antibodies against Cdc42, Rac1 or IQGAP1 corresponding to possible off-targets of ARHGEF9-directed siRNA. (TIF) [file ppat.1005307.s003.tif]

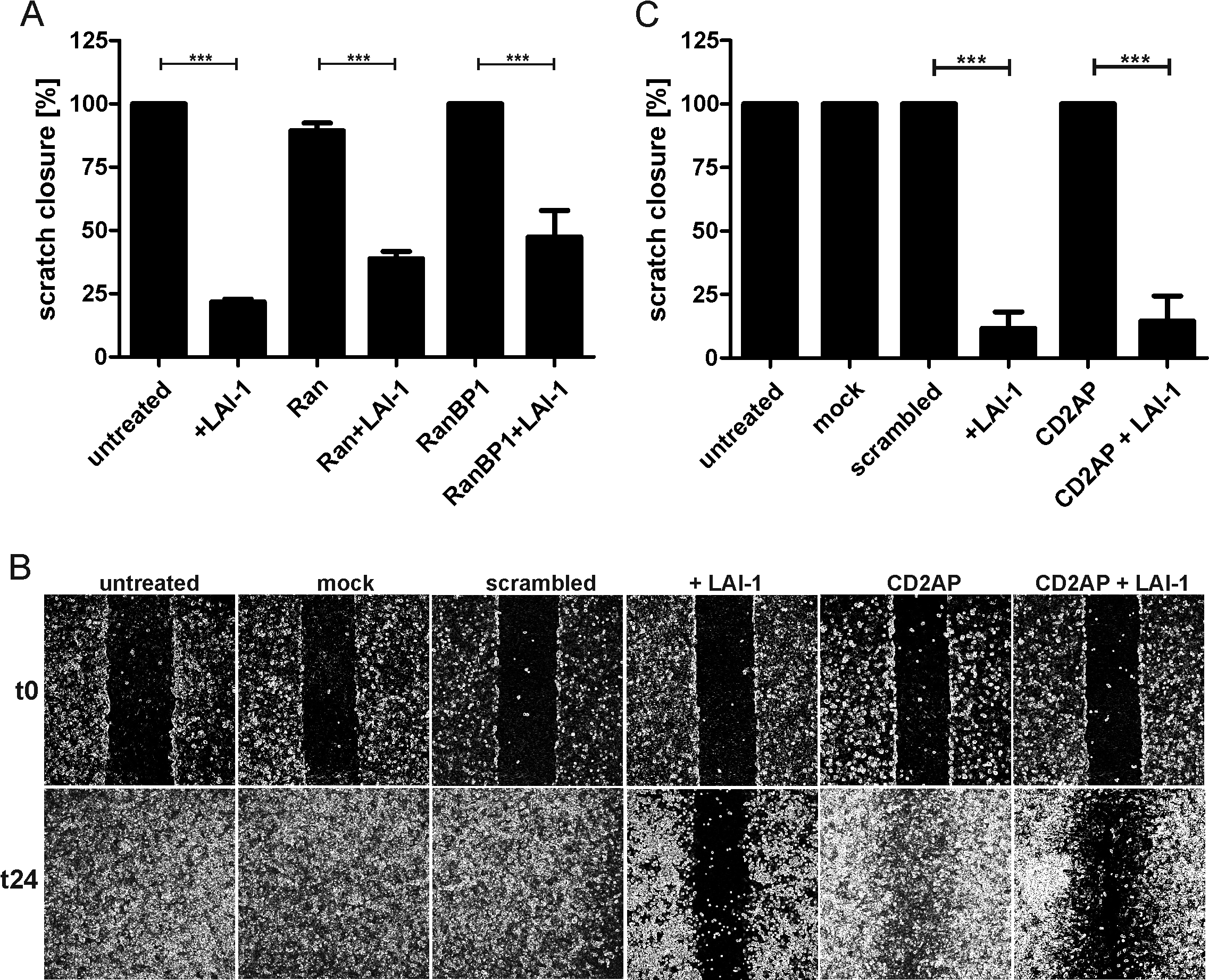

Supplement: S4 Fig — Confluent cell layers of A549 cells were left untreated or treated for 2 days with siRNA against (A) the small GTPase Ran or its effector RanBP1, or (B, C) the SH3-domain scaffold protein CDAP2, incubated with LAI-1 (10 μM, 1h) or not, scratched and let migrate for 24 h. Detached cells were washed off prior to imaging (0, 24 h). (A, C) The scratch area was quantified after 24 h using ImageJ software. Means and standard deviations of 3 independent experiments are shown (*** p < 0.001). The depletion efficiency of the siRNAs was assayed by Western blot (S3 Fig, [26]). (TIF) [file ppat.1005307.s004.tif]

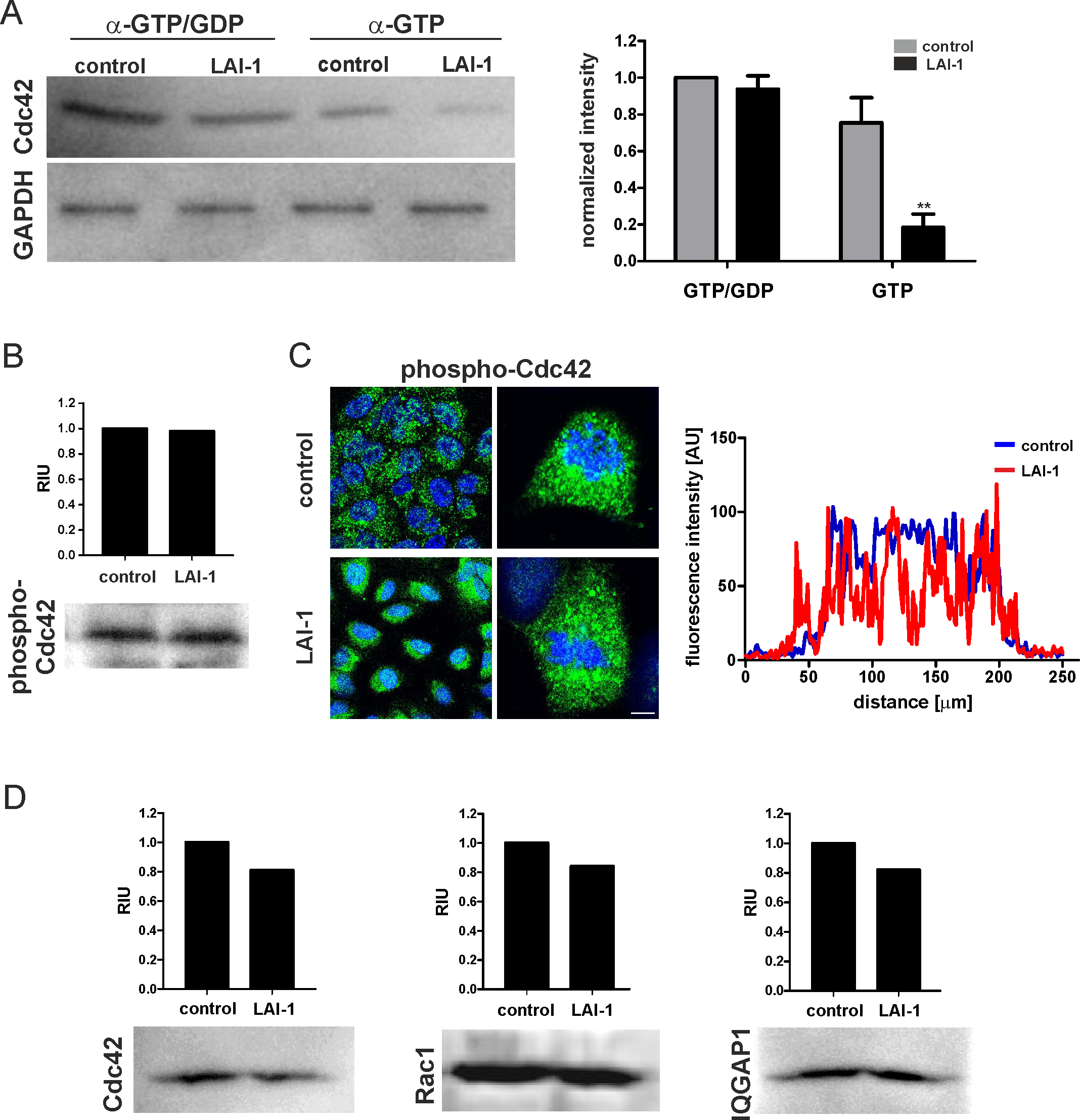

Supplement: S5 Fig — A549 cells were treated with LAI-1 (10 μM, 1 h) or not, and (A, B, D) lysed or (C) fixed. (A) Pull down with an antibody specifically recognizing Cdc42(GTP) and protein A/G agarose. The amount of active Cdc42 was analyzed by Western blot using an antibody recognizing Cdc42(GTP/GDP) (left panel). Quantification by densitometry was performed using ImageJ (right panel). Using an antibody against Cdc42/Rac1-phospho-Ser71 (B) Western blot or (C) immuno-fluorescence was performed (left panels: green, FITC; blue, DAPI; right panel: graph depicts the relative fluorescence intensity (arbitrary units, AU) along a section of a cell). Bar, 5 μm. (D) Western blots using antibodies against Cdc42, Rac1 or IQGAP1. (TIF) [file ppat.1005307.s005.tif]

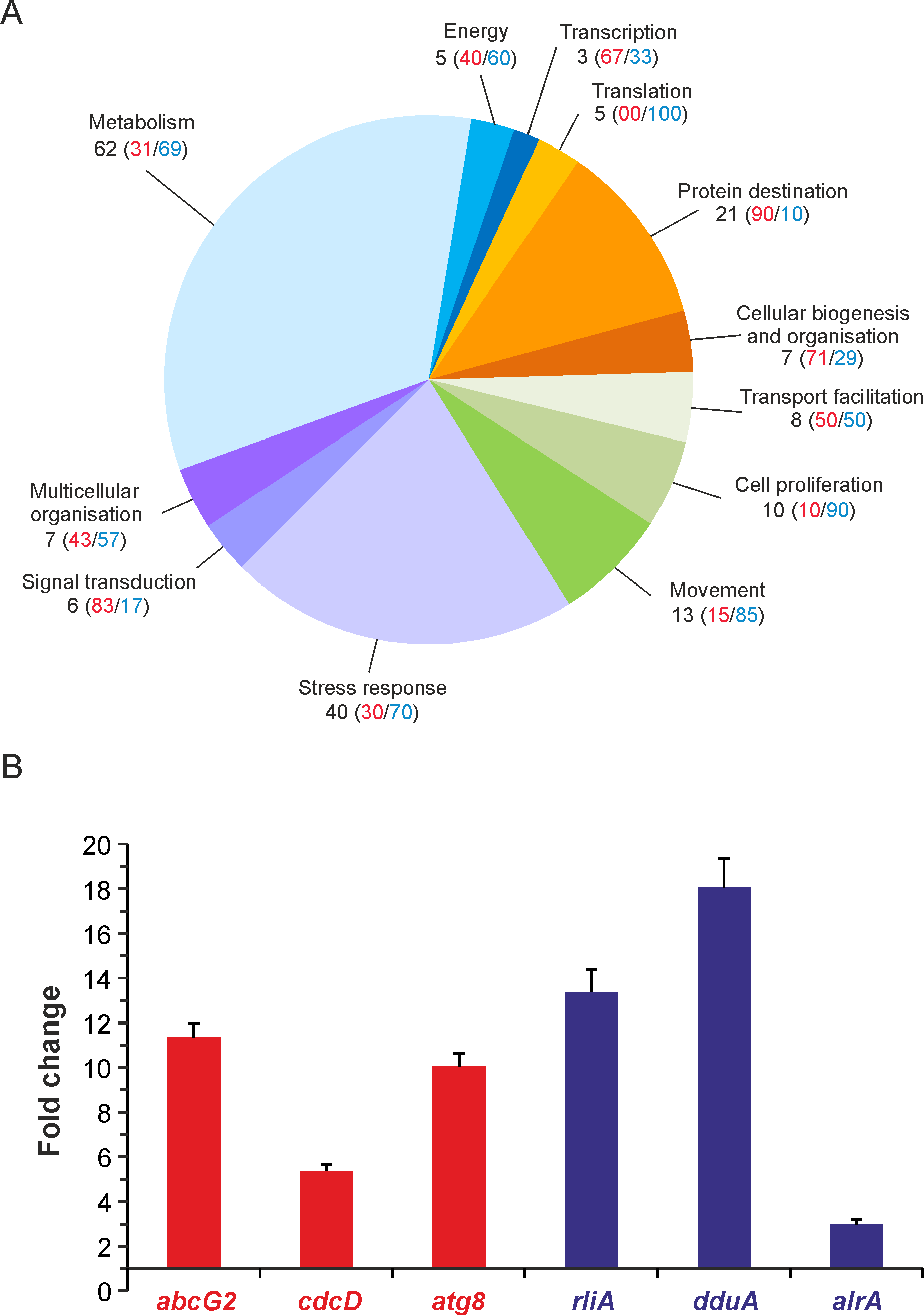

Supplement: S6 Fig — (A) Pie diagram of functionally categorised D. discoideum genes up- or down-regulated by 20 μM LAI-1. This concentration of LAI-1 led to robust changes in gene regulation, without being toxic to the amoebae. Shown are the absolute numbers of genes in different categories according to the yeast classification scheme and adapted to D. discoideum. Red and blue values in brackets indicate the percentage of up- and down-regulated genes in each category, respectively. (B) Validation of LAI-1-mediated differential expression of selected D. discoideum genes by quantitative real time (RT)-PCR using the oligonucleotides listed in S4 Table. The data indicate fold change in amoebae treated with 10 μM LAI-1 compared to control cells treated with DMSO only. Means and standard deviations of nine measurements from three independent RT-PCR experiments are shown. Red: up-regulated genes; blue: down-regulated genes. (TIF) [file ppat.1005307.s006.tif]

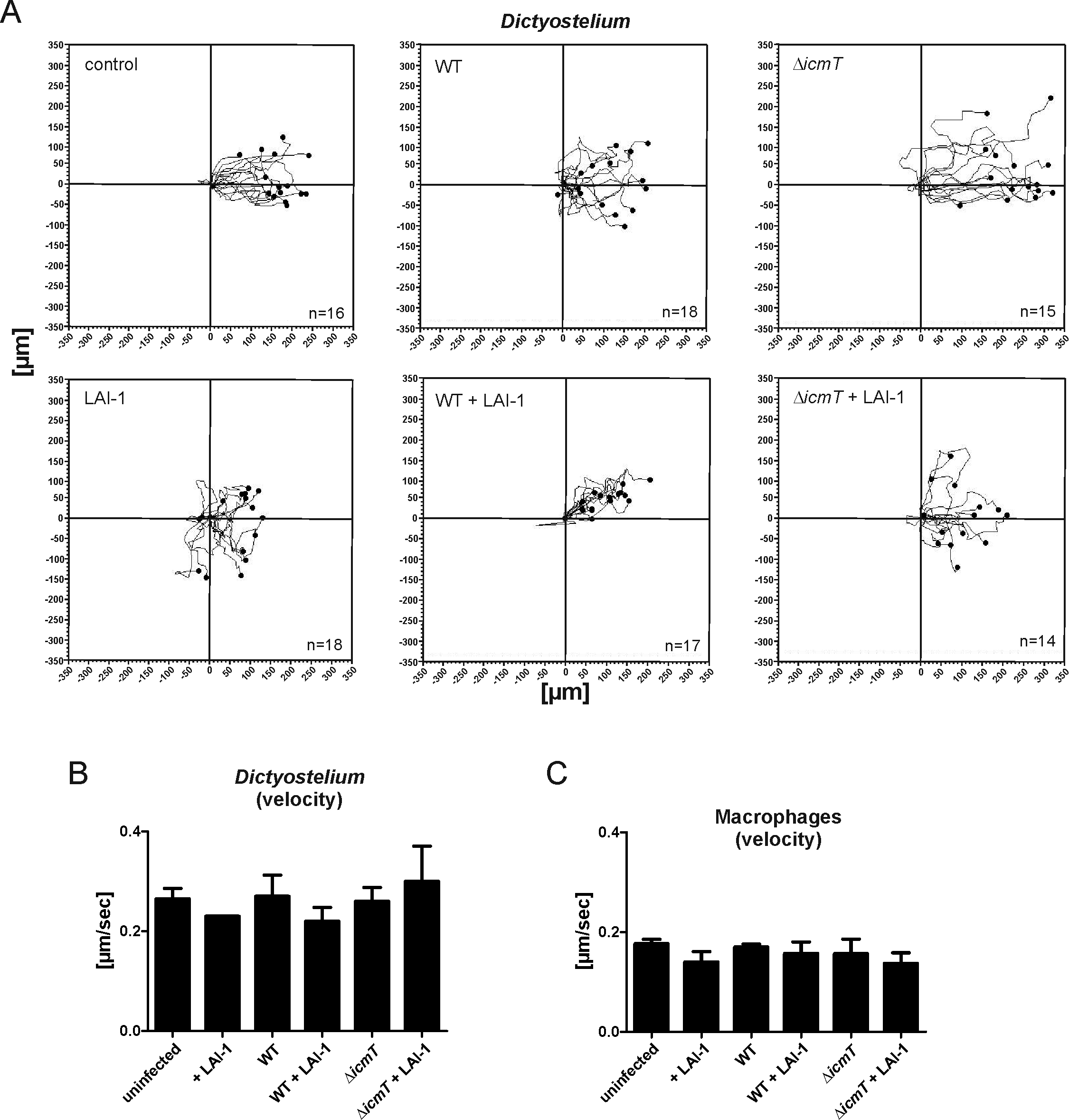

Supplement: S7 Fig — (A) D. discoideum Ax3 amoebae harboring pSW102 (GFP) or (C) RAW 264.7 macrophages were infected (MOI 10, 1 h) with L. pneumophila wild-type or ΔicmT mutant bacteria and treated with LAI-1 (10 μM, 1 h) or not. Single cell migration towards folate (1 mM) or CCL5 (100 ng/ml) was tracked in an under-agarose assay for 15 min or 1 h, respectively. (B, C) Motility parameters (velocity and forward migration index, FMI (Fig 7C)) were analyzed using the ImageJ manual tracker and Ibidi chemotaxis software. (TIF) [file ppat.1005307.s007.tif]

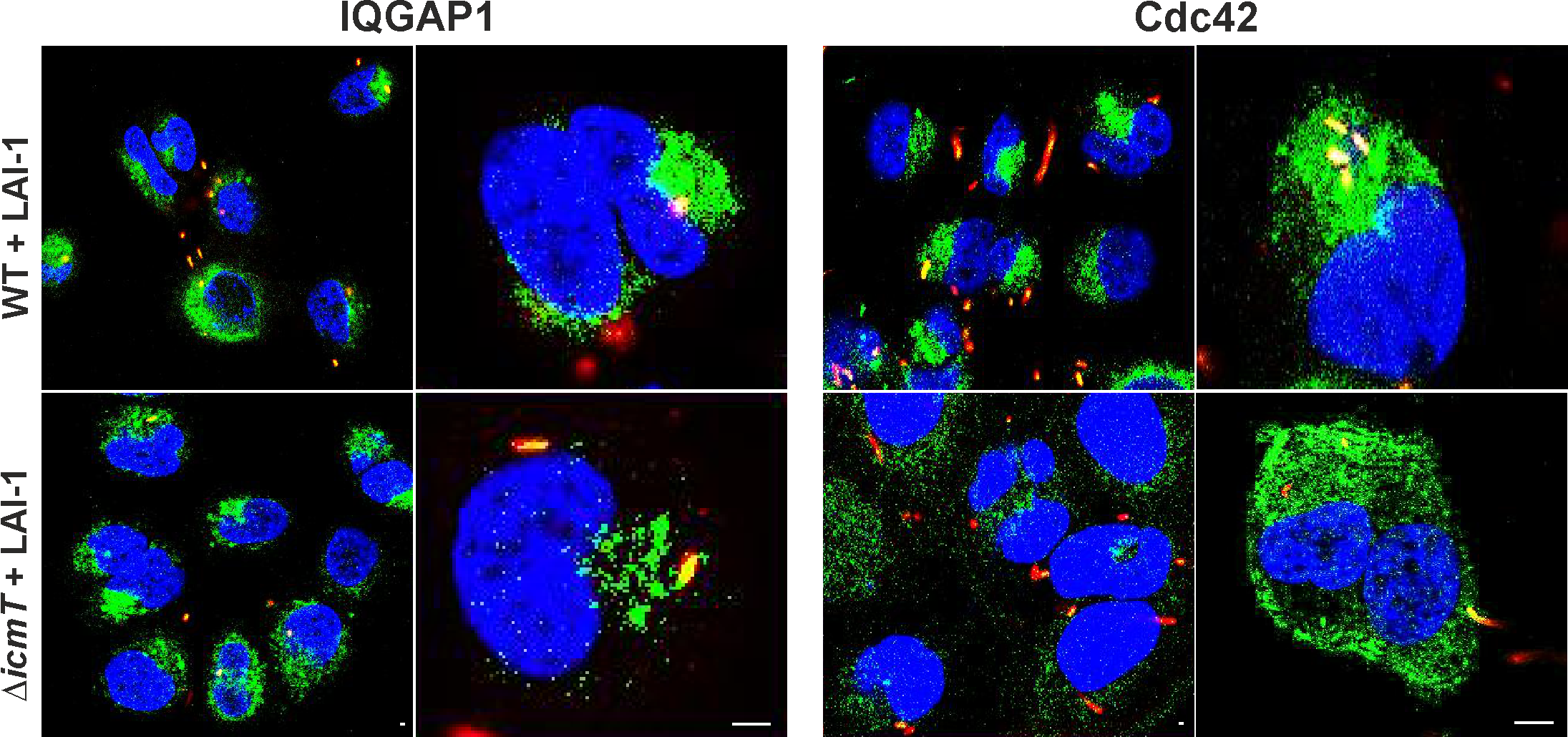

Supplement: S8 Fig — A549 cells were infected (MOI 10, 1 h) with L. pneumophila wild-type or ΔicmT mutant bacteria harboring plasmid pSW001 (DsRed) and treated with LAI-1 (10 μM, 1 h), fixed and stained with antibodies against IQGAP1 or Cdc42 (green). The cellular localization of IQGAP1 or Cdc42 was analyzed by confocal fluorescence microscopy (green, FITC; blue, DAPI). Bar: 5μm. (TIF) [file ppat.1005307.s008.tif]

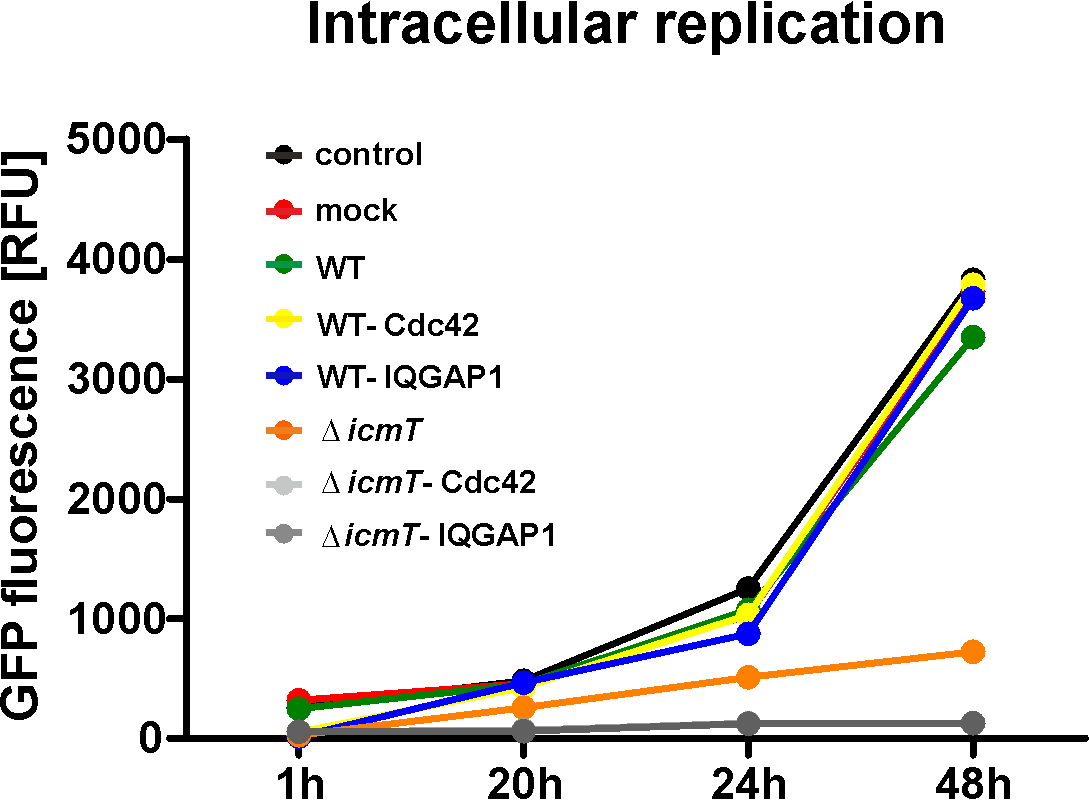

Supplement: S9 Fig — A549 cells were treated with a mixture of 4 different siRNAs against either Cdc42 or IQGAP1 for 2 days and infected with L. pneumophila wild-type or ΔicmT mutant bacteria harboring pCR76 (GFP). Fluorescence was measured at different timepoints post-infection (1, 20, 24 and 48 h). Depletion of Cdc42 or IQGAP1 does neither affect intracellular replication of wild-type L. pneumophila nor lack of replication of ΔicmT mutant bacteria. (TIF) [file ppat.1005307.s009.tif]
