# Supplementary material for: Inter-kingdom Signaling by the Legionella Quorum Sensing Molecule LAI-1 Modulates Cell Migration through an IQGAP1-Cdc42-ARHGEF9-Dependent Pathway
Source: PLoS Pathog. 2015 Dec 3;11(12):e1005307. doi: 10.1371/journal.ppat.1005307 (PMC4669118; doi:10.1371/journal.ppat.1005307)
Supplement: S1 Table — (DOCX) [file ppat.1005307.s010.docx]

**Table S1. Selected *D. discoideum* genes differentially regulated by LAI-1.**

| **DDB_G ID** | **FC** | **Gene Name** | **Gene Product** | **RT-PCR** |
| --- | --- | --- | --- | --- |
| DDB_G0274423 | **5,19** | DDB_G0274423 | SH3 domain-containing protein; homolog of CD2AP |  |
| DDB_G0275689 | **4,17** | abcG2 | ABC transporter G family protein | **yes** |
| DDB_G0288697 | **2,93** | DDB_G0288697 | Ubiquitin-conjugating enzyme family protein |  |
| DDB_G0293910 | **2,50** | DDB_G0293910 | ubiquitin superfamily protein |  |
| DDB_G0272566 | **2,47** | psmD14 | 26S proteasome non-ATPase regulatory subunit 14 |  |
| DDB_G0269206 | **2,27** | abcG21 | ABC transporter G family protein |  |
| DDB_G0288065 | **2,23** | cdcD | cell division cycle protein 48, CDC48 family AAA ATPase | **yes** |
| DDB_G0269462 | **2,12** | DDB_G0269462 | ubiquitin domain-containing protein |  |
| DDB_G0286191 | **2,10** | atg8 | autophagy protein 8 | **yes** |
| DDB_G0287461 | **2,10** | abcG3 | ABC transporter G family protein |  |
| DDB_G0275323 | **1,70** | tipD | autophagy protein 16 |  |
|  |  |  |  |  |
| DDB_G0272783 | **0,28** | rliA | major facilitator superfamily protein | **yes** |
| DDB_G0282559 | **0,32** | dduA | metallophosphoesterase domain-containing protein | **yes** |
| DDB_G0293850 | **0,35** | alrA | aldehyde reductase, aldo-keto reductase | **yes** |
| DDB_G0273175 | **0,35** | cf50-2 | component of the CF complex |  |
| DDB_G0285025 | **0,36** | alrE | aldo-keto reductase |  |
| DDB_G0288563 | **0,40** | DDB_G0288563 | cysteine proteinase |  |
| DDB_G0281605 | **0,41** | cfaD | countin factor associated protein |  |
| DDB_G0282153 | **0,41** | aplH | amoebapore-like protein H |  |
| DDB_G0280531 | **0,41** | tgrC1 | tiger protein C1 |  |
| DDB_G0278721 | **0,41** | cprD | cysteine proteinase 4 |  |
| DDB_G0275693 | **0,42** | DDB_G0275693 | peptidase C53 family protein |  |
| DDB_G0280187 | **0,42** | DDB_G0280187 | peptidase C1A family protein |  |
| DDB_G0279187 | **0,45** | cprG | cysteine proteinase 7 |  |
| DDB_G0293014 | **0,47** | DDB_G0293014 | serine protease, peptidase S28 family protein |  |
| DDB_G0283401 | **0,47** | ctsZ | peptidase C1A family protein |  |
| DDB_G0274551 | **0,48** | rcdBB | similar to lysozyme-like protein 4 precursor |  |
| DDB_G0274597 | **0,50** | ctnA | component of the CF complex, countin A |  |

FC: Fold change; red: up-regulated; blue: down-regulated; CF: counting factor
