# Supplementary material for: Inter-kingdom Signaling by the Legionella Quorum Sensing Molecule LAI-1 Modulates Cell Migration through an IQGAP1-Cdc42-ARHGEF9-Dependent Pathway
Source: PLoS Pathog. 2015 Dec 3;11(12):e1005307. doi: 10.1371/journal.ppat.1005307 (PMC4669118; doi:10.1371/journal.ppat.1005307)
Supplement: S2 Table — (DOCX) [file ppat.1005307.s011.docx]

**Table S2. All *D. discoideum* genes differentially regulated by LAI-1.**

| **Up-regulated genes**^a^ | | | |
| --- | --- | --- | --- |
| **DDB_G ID** | **FC** | **Gene Name** | **Gene Product** |
| DDB_G0274423 | 5,19 | DDB_G0274423 | SH3 domain-containing protein; homolog of CD2-associated protein [*Xenopus laevis*]; NP_001086432.1 |
| DDB_G0275689 | 4,17 | abcG2 | ABC transporter G family protein |
| DDB_G0274289 | 3,49 | DDB_G0274289 | unknown |
| DDB_G0268874 | 3,09 | DDB_G0268874 | unknown |
| DDB_G0282075 | 2,94 | xpnpep3 | peptidase M24 family protein, Xaa-Pro aminopeptidase 3 |
| DDB_G0288697 | 2,93 | DDB_G0288697 | ubiquitin-conjugating enzyme family protein |
| DDB_G0276951 | 2,73 | hspG7 | heat shock protein Hsp20 domain-containing protein |
| DDB_G0274339 | 2,68 | DDB_G0274339 | acetyl-CoA C-acyltransferase, beta-ketothiolase,  3-ketoacyl-CoA thiolase |
| DDB_G0293202 | 2,50 | DDB_G0293202 | TRAF-type zinc finger-containing protein, meprin and TRAF homology (MATH) domain-containing protein |
| DDB_G0293910 | 2,50 | DDB_G0293910 | ubiquitin superfamily protein |
| DDB_G0286239 | 2,49 | DDB_G0286239 | α/βhydrolase fold-1 domain-containing protein, serine hydrolase-like protein |
| DDB_G0272566 | 2,47 | psmD14 | 26S proteasome non-ATPase regulatory subunit 14, putative multidrug resistance protein |
| DDB_G0285899 | 2,47 | DDB_G0285899 | alanine transaminase, glutamate pyruvate transaminase |
| DDB_G0279159 | 2,40 | aco1 | putative iron regulatory protein, aconitate hydratase, aconitase |
| DDB_G0290377 | 2,39 | agnB | argonaut-like protein |
| DDB_G0278865 | 2,39 | DDB_G0278865 | unknown |
| DDB_G0272720 | 2,33 | DDB_G0272720 | unknown |
| DDB_G0276361 | 2,27 | DDB_G0276361 | unknown |
| DDB_G0291281 | 2,27 | DDB_G0291281 | thioredoxin-like superfamily; glutaredoxin-like protein |
| DDB_G0269206 | 2,27 | abcG21 | ABC transporter G family protein |
| DDB_G0280919 | 2,24 | DDB_G0280919 | unknown |
| DDB_G0288065 | 2,23 | cdcD | cell division cycle protein 48, CDC48 family AAA ATPase |
| DDB_G0277849 | 2,20 | dymA | dynamin A |
| DDB_G0272724 | 2,19 | DDB_G0272724 | contains several VQPM repeats and long stretches of His |
| DDB_G0270012 | 2,17 | DDB_G0270012 | unknown |
| DDB_G0288651 | 2,13 | DDB_G0288651 | patatin-like phospholipase family glycoprotein; (storage protein, lipase) |
| DDB_G0269462 | 2,12 | DDB_G0269462 | ubiquitin domain-containing protein |
| DDB_G0286191 | 2,10 | atg8 | autophagy protein 8 |
| DDB_G0287461 | 2,10 | abcG3 | ABC transporter G family protein |
| DDB_G0291798 | 2,09 | DDB_G0291798 | unknown |
| DDB_G0278163 | 2,06 | DDB_G0278163 | TM2 domain-containing protein |
| DDB_G0285615 | 2,05 | iliA | unknown (induced after *Legionella* infection) |
| DDB_G0280211 | 2,02 | DDB_G0280211 | unknown |
| DDB_G0287993 | 2,00 | vps60 | SNF7 family protein |
| DDB_G0272280 | 1,98 | DDB_G0272280 | thioredoxin-like superfamily; AhpC/TSA family protein |
| DDB_G0290723 | 1,95 | mkcB | putative protein serine/threonine kinase, STE20 family protein kinase, MKC subfamily protein kinase |
| DDB_G0281781 | 1,94 | DDB_G0281781 | unknown |
| DDB_G0282061 | 1,93 | DDB_G0282061 | unknown |
| DDB_G0284347 | 1,91 | vps4 | AAA ATPase domain-containing protein, MIT domain-containing protein |
| DDB_G0278115 | 1,91 | netD | nuclear envelope transmembrane protein 4 |
| DDB_G0272833 | 1,90 | csbA | contact site B protein |
| DDB_G0280471 | 1,90 | DDB_G0280471 | unknown |
| DDB_G0282905 | 1,90 | DDB_G0282905 | transmembrane protein |
| DDB_G0270884 | 1,87 | abnA | actobindin A |
| DDB_G0281653 | 1,86 | DDB_G0281653 | unknown |
| DDB_G0289327 | 1,85 | sevA | severin |
| DDB_G0283937 | 1,84 | sec1 | Sec1-like family protein |
| DDB_G0267402 | 1,84 | H3a | histone H3 |
| DDB_G0277491 | 1,84 | hspG12 | heat shock protein Hsp20 domain-containing protein, putative α-crystallin-type heat shock protein |
| DDB_G0267394 | 1,83 | vps46 | SNF7 family protein |
| DDB_G0273017 | 1,82 | DDB_G0273017 | isocitrate lyase |
| DDB_G0269112 | 1,81 | celB | cellulose-binding protein |
| DDB_G0270990 | 1,81 | DDB_G0270990 | putative acyl-CoA oxidase |
| DDB_G0277119 | 1,81 | hspG8 | heat shock protein Hsp20 domain-containing protein |
| DDB_G0281913 | 1,81 | DDB_G0281913 | unknown |
| DDB_G0270362 | 1,81 | zfand | AN1-type zinc finger protein |
| DDB_G0276287 | 1,80 | expl3 | expansin-like protein |
| DDB_G0283253 | 1,79 | DDB_G0283253 | unknown |
| DDB_G0288203 | 1,78 | DDB_G0288203 | armadillo repeat protein; N-terminal region similar to the IFN-related developmental regulators; one HEAT repeat |
| DDB_G0271488 | 1,78 | vta1 | unknown |
| DDB_G0271376 | 1,78 | DDB_G0271376 | nudix hydrolase superfamily; ADPRase_NUDT5 domain: ADP-ribose pyrophosphatase hydrolyses ADP-ribose and other ADP-sugar conjugates to AMP and ribose-5-P |
| DDB_G0272514 | 1,78 | DDB_G0272514 | unknown |
| DDB_G0269482 | 1,78 | DDB_G0269482 | unknown |
| DDB_G0293416 | 1,78 | abcB1 | ABC transporter B family protein |
| DDB_G0271294 | 1,77 | DDB_G0271294 | similar to E3 ubiquitin-protein ligase RNF181-like |
| DDB_G0267426 | 1,77 | cshA | citrate synthase |
| DDB_G0287717 | 1,76 | iliD | putative phytanoyl-CoA dioxygenase |
| DDB_G0279527 | 1,74 | DDB_G0279527 | unknown |
| DDB_G0292116 | 1,74 | DDB_G0292116 | glucose/ribitol dehydrogenase family protein |
| DDB_G0270098 | 1,74 | sqstm1 | sequestosome-1 |
| DDB_G0275573 | 1,74 | vps32 | SNF7 family protein |
| DDB_G0272182 | 1,72 | DDB_G0272182 | putative arginine deiminase |
| DDB_G0287841 | 1,71 | DDB_G0287841 | unknown |
| DDB_G0277429 | 1,70 | DDB_G0277429 | regulator of chromosome condensation (RCC1) domain-containing protein, BTB/POZ domain-containing protein |
| DDB_G0288935 | 1,70 | panC | pantoate-β-alanine ligase |
| DDB_G0275323 | 1,70 | tipD | autophagy protein 16 |
| DDB_G0276817 | 1,69 | DDB_G0276817 | unknown |
| DDB_G0272350 | 1,68 | sgkC | sphingosine kinase related protein |
| DDB_G0274291 | 1,68 | DDB_G0274291 | putative T4-like lysozyme, glycoside hydrolase family 24 |
| DDB_G0272098 | 1,66 | DDB_G0272098 | TNF receptor-associated factor 5 |
| DDB_G0291518 | 1,66 | DDB_G0291518 | putative transmembrane protein |
| DDB_G0278285 | 1,66 | copB2 | WD40 repeat-containing protein, coatomer protein complex β2 (β) subunit |
| DDB_G0278807 | 1,65 | DDB_G0278807 | unknown |
| DDB_G0270414 | 1,65 | tmem56C | TRAM, LAG1 and CLN8 homology domain-containing protein, TMEM56 family protein 3 |
| DDB_G0278013 | 1,64 | DDB_G0278013 | unknown |
| DDB_G0279973 | 1,64 | DDB_G0279973 | thioredoxin-like superfamily; AhpC/TSA family protein |
| DDB_G0269176 | 1,64 | racF1 | Rho GTPase |
| DDB_G0284831 | 1,60 | 4cl1 | 4-coumarate-CoA ligase |
| DDB_G0282197 | 1,60 | DDB_G0282197 | membrane bound O-acyl transferase family protein |
| DDB_G0289397 | 1,60 | DDB_G0289397 | unknown |
| DDB_G0284777 | 1,60 | DDB_G0284777 | DUF1183 family protein; similar to store-operated calcium entry-associated regulatory factor |
| DDB_G0269174 | 1,57 | rab1C | Rab GTPase |
| DDB_G0284841 | 1,57 | DDB_G0284841 | unknown |
| DDB_G0289029 | 1,57 | DDB_G0289029 | regulator of Vps4 activity in the MVB pathway; similar to *S. cerevisiae* IST1 (increased sodium tolerance) involved in endosome to vacuole transport via the MVB body sorting pathway; N-terminal DUF292 domain |
| DDB_G0270838 | 1,57 | H3b | histone H3 |
| DDB_G0285767 | 1,56 | DDB_G0285767 | unknown |
| DDB_G0271264 | 1,56 | usp14 | peptidase C19 family ubiquitin domain-containing protein, putative ubiquitin carboxyl-terminal hydrolase (UCH) |
| DDB_G0277097 | 1,55 | uduF | unknown |
| DDB_G0268092 | 1,55 | DDB_G0268092 | similar to human zinc finger SWIM domain protein 7, regulator of homologous recombination in eukaryotic cells |
| DDB_G0271340 | 1,55 | DDB_G0271340 | acyl-CoA oxidase |
| DDB_G0286143 | 1,54 | DDB_G0286143 | dullard-like phosphatase domain containing protein; TFIIF-interacting CTD phosphatases, including NLI-interacting factor (transcription) |
| DDB_G0276769 | 1,54 | DDB_G0276769 | unknown |
| DDB_G0276789 | 1,54 | DDB_G0276789 | unknown |
| DDB_G0287671 | 1,53 | DDB_G0287671 | prolyl 4-hydroxylase homolog; prolyl hydroxylase is a member of the 2-oxoglutarate-dependent dioxygenases |
| DDB_G0291834 | 1,53 | cpiA | cystatin A1, cysteine protease inhibitor |
| DDB_G0291155 | 1,53 | DDB_G0291155 | RNA-binding region RNP-1 domain-containing protein, RNA recognition motif-containing protein RRM |
| DDB_G0267456 | 1,53 | cbp2 | calcium-binding protein |
| DDB_G0281983 | 1,52 | tmem50 | UPF0220 family TM protein, TMEM50 family protein |
| DDB_G0270978 | 1,52 | DDB_G0270978 | putative transmembrane protein |
| DDB_G0276383 | 1,52 | DDB_G0276383 | putative guanylate cyclase |
| DDB_G0270104 | 1,51 | DDB_G0270104 | putative NADH dehydrogenase |
| DDB_G0287741 | 1,51 | DDB_G0287741 | enoyl-CoA hydratase/isomerase domain-containing protein |
| DDB_G0274595 | 1,51 | catA | catalase |
| DDB_G0275209 | 1,51 | cnrD | ubiquitin system component Cue domain-containing protein, putative cell number regulator |
| DDB_G0273289 | 1,50 | DDB_G0273785 | unknown |

| **Down-regulated genes**^a^ | | | |
| --- | --- | --- | --- |
| **DDB_G ID** | **FC** | **Gene Name** | **Gene Product** |
| DDB_G0272783 | 0,28 | rliA | major facilitator superfamily protein |
| DDB_G0282559 | 0,32 | dduA | metallophosphoesterase domain-containing protein, N-terminal purple acid phosphatase domain protein |
| DDB_G0278725 | 0,34 | p17 | unknown |
| DDB_G0293850 | 0,35 | alrA | aldehyde reductase, aldo-keto reductase |
| DDB_G0273175 | 0,35 | cf50-2 | GH25_muramidase superfamily; component of the counting factor (CF) complex |
| DDB_G0285025 | 0,36 | alrE | aldo-keto reductase |
| DDB_G0275007 | 0,40 | cmfA | conditioned medium factor, density sensing factor |
| DDB_G0281399 | 0,40 | DDB_G0281399 | unknown |
| DDB_G0288563 | 0,40 | DDB_G0288563 | cysteine proteinase |
| DDB_G0281605 | 0,41 | cfaD | counting factor-associated protein, cathepsin L-like proteinase, peptidase C1A family protein |
| DDB_G0282153 | 0,41 | aplH | amoebapore-like protein H |
| DDB_G0280531 | 0,41 | tgrC1 | tiger protein C1 |
| DDB_G0278721 | 0,41 | cprD | cysteine proteinase 4 |
| DDB_G0275693 | 0,42 | DDB_G0275693 | peptidase C53 family protein |
| DDB_G0282517 | 0,42 | DDB_G0282517 | AhpC/TSA family protein; thioredoxin_like superfamily |
| DDB_G0280187 | 0,42 | DDB_G0280187 | peptidase C1A family protein, papain family cysteine protease, cathepsin Z-like protein |
| DDB_G0291255 | 0,43 | 29C | unknown |
| DDB_G0268156 | 0,44 | DDB_G0268156 | unknown |
| DDB_G0284671 | 0,44 | DDB_G0284671 | unknown |
| DDB_G0288481 | 0,44 | gchA | GTP cyclohydrolase I |
| DDB_G0280317 | 0,44 | DDB_G0280317 | putative glutathione S-transferase, putative glutathione transferase |
| DDB_G0293122 | 0,45 | DDB_G0293122 | glutathione S-transferase, glutathione transferase |
| DDB_G0279187 | 0,45 | cprG | cysteine proteinase 7 |
| DDB_G0279437 | 0,46 | DDB_G0279437 | cytochrome b561/ferric reductase transmembrane domain-containing protein, DOMON domain-containing protein |
| DDB_G0292206 | 0,46 | manA | α-mannosidase |
| DDB_G0275069 | 0,46 | pks16 | putative fatty acid synthase |
| DDB_G0293014 | 0,47 | DDB_G0293014 | serine protease, peptidase S28 family protein |
| DDB_G0283401 | 0,47 | ctsZ | cathepsin Z precursor, peptidase C1A family protein, papain family cysteine protease |
| DDB_G0285341 | 0,47 | DDB_G0285341 | unknown |
| DDB_G0273411 | 0,48 | DDB_G0273555 | elongation factor P |
| DDB_G0267570 | 0,48 | DDB_G0267570 | unknown |
| DDB_G0274551 | 0,48 | rcdBB | similar to lysozyme-like protein 4 precursor [*Rattus norvegicus*] |
| DDB_G0284579 | 0,49 | DDB_G0284579 | putative transporter |
| DDB_G0271892 | 0,49 | DDB_G0271892 | putative glutathione S-transferase |
| DDB_G0288907 | 0,50 | smtA | putative δ-24-sterol methyltransferase Smt1 |
| DDB_G0274597 | 0,50 | ctnA | component of the counting factor (CF) complex, countin |
| DDB_G0277729 | 0,50 | DDB_G0277729 | unknown |
| DDB_G0281293 | 0,50 | ddiA | ribonuclease T2 |
| DDB_G0279411 | 0,51 | ctsD | cathepsin D, preprocathepsin D |
| DDB_G0279921 | 0,51 | gp130 | glycoprotein 130, required for growth on Gram^+^ bacteria |
| DDB_G0289393 | 0,52 | psiH | PA14 domain-containing protein |
| DDB_G0293874 | 0,52 | mcfZ | mitochondrial substrate carrier family protein |
| DDB_G0287363 | 0,52 | sibA | integrin beta-like protein A |
| DDB_G0274451 | 0,52 | DDB_G0274451 | unknown |
| DDB_G0290537 | 0,52 | DDB_G0290537 | putative NAD-dependent aldehyde dehydrogenase |
| DDB_G0286561 | 0,52 | aplC | amoebapore-like protein C |
| DDB_G0273837 | 0,52 | scd2-2 | δ-9 fatty acid desaturase Scd2 |
| DDB_G0292250 | 0,52 | sqrdl | putative sulfide quinone reductase |
| DDB_G0291191 | 0,52 | DDB_G0291191 | cysteine protease |
| DDB_G0272815 | 0,52 | cprE | cysteine proteinase 5 |
| DDB_G0267386 | 0,53 | cysB | cystathionine β-synthase |
| DDB_G0280105 | 0,53 | DDB_G0280105 | peptidase S10 family protein, serine carboxypeptidase |
| DDB_G0283095 | 0,53 | DD3-3 | unknown |
| DDB_G0278793 | 0,55 | DDB_G0278793 | regulator of microtubule dynamics protein 1 |
| DDB_G0283987 | 0,55 | purC/E | phosphoribosylaminoimidazole carboxylase (AIR carboxylase), phosphoribosylaminoimidazolesuccino-carboxamide synthase (SAICAR synthase), PAICS |
| DDB_G0286651 | 0,56 | aplB | amoebapore-like protein B |
| DDB_G0274465 | 0,56 | DDB_G0274465 | DrsE domain containing protein; DsrE is a small soluble protein involved in intracellular sulfur reduction. |
| DDB_G0281551 | 0,56 | guaA | GMP synthetase |
| DDB_G0281403 | 0,56 | nacA | putative nascent polypeptide-associated complex α subunit |
| DDB_G0290603 | 0,56 | DDB_G0290603 | unknown |
| DDB_G0275359 | 0,57 | DDB_G0275359 | putative S-adenosylmethionine-dependent methyltransferase |
| DDB_G0292986 | 0,57 | abcG10 | ABC transporter G family protein |
| DDB_G0289157 | 0,57 | cmfB | putative CMF receptor CMFR1 |
| DDB_G0294589 | 0,57 | DDB_G0286849 | short-chain dehydrogenase/reductase (SDR) family protein, glucose/ribitol dehydrogenase family protein |
| DDB_G0283701 | 0,58 | guaB | IMP (inosine-5'-monophosphate) dehydrogenase |
| DDB_G0276335 | 0,58 | pyr1-3 | dihydroorotase, aspartate carbamoyltransferase, glutamine-dependent carbamoyl-phosphate synthase |
| DDB_G0287033 | 0,58 | nagA | β-N-acetylhexosaminidase, glycoside hydrolase family 20 protein, β-hexosaminidase |
| DDB_G0270260 | 0,58 | nhp6 | HMG1/2 (high mobility group) box-containing protein |
| DDB_G0267380 | 0,58 | argE | acetylornithine deacetylase |
| DDB_G0292378 | 0,58 | fhbA | flavohemoglobin |
| DDB_G0269248 | 0,58 | cf45-1 | component of the counting factor (CF) complex; glycoside hydrolase family 25 protein |
| DDB_G0275525 | 0,58 | hspK | heat shock protein Hsp20 domain-containing protein, putative alpha-crystallin-type heat shock protein |
| DDB_G0288677 | 0,59 | cdk5 | cyclin-dependent kinase 5 |
| DDB_G0282143 | 0,59 | hatB | actin binding protein, hisactophilin II |
| DDB_G0278341 | 0,59 | DDB_G0278341 | putative ATP citrate synthase, putative ATP citrate lyase |
| DDB_G0276279 | 0,59 | DDB_G0276279 | probable iron/ascorbate oxidoreductase |
| DDB_G0295807 | 0,59 | DDB_G0295807 | short-chain dehydrogenase/reductase (SDR) family protein, glucose/ribitol dehydrogenase family protein |
| DDB_G0275333 | 0,60 | DDB_G0275333 | ARID/BRIGHT DNA binding domain-containing protein |
| DDB_G0293124 | 0,60 | kxcB | putative protein serine/threonine kinase (STE group), RhoGEF domain-containing protein, DH domain-containing protein, PH domain-containing protein |
| DDB_G0269144 | 0,60 | hspB | heat shock protein, heat shock cognate protein Hsc70-1 |
| DDB_G0277855 | 0,60 | fimA | fimbrin-1 |
| DDB_G0288333 | 0,60 | purB | adenylosuccinate lyase |
| DDB_G0287607 | 0,60 | pcna | proliferating cell nuclear antigen |
| DDB_G0288579 | 0,60 | DDB_G0288579 | unknown |
| DDB_G0275227 | 0,60 | DDB_G0275227 | FKBP-type peptidylprolyl cis-trans isomerase (PPIase) |
| DDB_G0269216 | 0,60 | cypE | cyclophilin-type peptidylprolyl cis-trans isomerase (PPIase) |
| DDB_G0267400 | 0,60 | hspD | heat shock protein Hsp90 family protein |
| DDB_G0269940 | 0,61 | osbC | oxysterol binding family protein, member 3 |
| DDB_G0275911 | 0,61 | DDB_G0275911 | kelch repeat-containing protein |
| DDB_G0287127 | 0,61 | vatA | vacuolar ATPase subunit A |
| DDB_G0270922 | 0,61 | DDB_G0270922 | unknown |
| DDB_G0280057 | 0,61 | grp94 | heat shock protein Hsp90 family protein |
| DDB_G0290659 | 0,61 | sdrA | short-chain dehydrogenase/reductase (SDR) family protein |
| DDB_G0286725 | 0,61 | vps13A | vacuolar protein sorting-associated protein 13 family protein |
| DDB_G0279925 | 0,61 | mlkA | myosin light chain kinase A |
| DDB_G0281283 | 0,62 | DDB_G0281283 | probable N-acetyltransferase |
| DDB_G0282371 | 0,62 | veg111 | hyaluronidase |
| DDB_G0291121 | 0,62 | cinB | esterase/lipase/thioesterase domain-containing protein, vegetative specific protein H5 |
| DDB_G0282077 | 0,62 | DDB_G0282077 | unknown |
| DDB_G0287139 | 0,62 | DDB_G0287139 | unknown |
| DDB_G0288879 | 0,62 | act11 | actin |
| DDB_G0280533 | 0,63 | lmcB | unknown |
| DDB_G0281045 | 0,63 | DDB_G0281045 | paired amphipathic helix (PAH) containing protein |
| DDB_G0268948 | 0,63 | DDB_G0268948 | putative SAM dependent methyltransferase |
| DDB_G0291912 | 0,63 | DDB_G0291912 | peptidase S10 family protein, serine carboxypeptidase, carboxypeptidase C |
| DDB_G0277947 | 0,63 | shmt1 | serine hydroxymethyltransferase 1 |
| DDB_G0277087 | 0,63 | purH | AICAR transformylase IMP cyclohydrolase, inosine monophosphate cyclohydrolase/phosphoribosylamino-imidazolecarboxamide formyltransferase |
| DDB_G0278345 | 0,63 | acly | ATP citrate synthase, ATP citrate lyase |
| DDB_G0272186 | 0,64 | DDB_G0272186 | tetratricopeptide-like helical domain-containing protein (TPR), TPR_1 repeat-containing protein |
| DDB_G0287335 | 0,64 | DDB_G0287335 | RNA-binding region RNP-1 domain-containing protein, RNA recognition motif-containing protein RRM |
| DDB_G0269928 | 0,64 | DDB_G0269928 | DUF3430 family protein |
| DDB_G0285323 | 0,64 | atg9 | autophagy protein 9 |
| DDB_G0284363 | 0,64 | cf60 | component of the counting factor (CF) complex, histidine acid phosphatase family protein |
| DDB_G0272795 | 0,64 | 3B-1 | prespore-specific protein |
| DDB_G0267454 | 0,64 | ancA | ADP/ATP translocase, transmembrane protein, mitochondrial substrate carrier family protein |
| DDB_G0287277 | 0,64 | DDB_G0287277 | NAD-dependent epimerase/dehydratase family protein |
| DDB_G0288715 | 0,65 | gadB | glutamate decarboxylase B |
| DDB_G0291179 | 0,65 | metK | S-adenosylmethionine synthetase |
| DDB_G0270488 | 0,65 | DDB_G0270488 | laminin egf-like (domains iii and v) domain protein |
| DDB_G0292388 | 0,65 | rpl27a | S60 ribosomal protein L27a |
| DDB_G0267382 | 0,65 | corA | coronin |
| DDB_G0269196 | 0,65 | tubB | βtubulin |
| DDB_G0286057 | 0,65 | adk | adenosine kinase, ATP:adenosine 5'-phosphotransferase |
| DDB_G0283915 | 0,65 | DDB_G0283915 | esterase/lipase/thioesterase domain-containing protein |
| DDB_G0272106 | 0,65 | arpB | actin related protein 2, polyphosphate kinase component |
| DDB_G0278581 | 0,65 | psiF | discoidin-inducing complex (DIC) PA14 domain protein |
| DDB_G0269016 | 0,65 | cyp524A1 | cytochrome P450 family protein |
| DDB_G0273805 | 0,65 | ndkC-2 | nucleoside diphosphate kinase, NDP kinase |
| DDB_G0271668 | 0,65 | rpl36 | S60 ribosomal protein L36 |
| DDB_G0272861 | 0,66 | cosA | actin regulatory protein costars |
| DDB_G0293898 | 0,66 | coaA | actin binding protein, coactosin |
| DDB_G0295779 | 0,66 | DDB_G0295779 | EGF-like domain-containing protein |
| DDB_G0269890 | 0,66 | DDB_G0269890 | YjeF N-terminal domain-containing protein |
| DDB_G0280319 | 0,66 | glud2 | NAD^+^ dependent glutamate dehydrogenase |
| DDB_G0288131 | 0,66 | cmbB | calmodulin-binding protein CmbB |
| DDB_G0280159 | 0,66 | DDB_G0280159 | unknown |
| DDB_G0276049 | 0,66 | DDB_G0276049 | unknown |
| DDB_G0281447 | 0,66 | DDB_G0281447 | unknown |
| DDB_G0292804 | 0,66 | arcC | actin related protein 2/3 complex, subunit 3 (21 kDa) |
| DDB_G0274113 | 0,66 | rpl8 | 60S ribosomal protein L8 |
| DDB_G0275559 | 0,66 | DDB_G0275559 | short-chain dehydrogenase/reductase (SDR) family protein, glucose/ribitol dehydrogenase family protein |
| DDB_G0289025 | 0,66 | rps7 | 40S ribosomal protein S7 |
| DDB_G0274321 | 0,66 | purF | phosphoribosylpyrophosphate amidotransferase |
| DDB_G0290333 | 0,66 | DDB_G0290333 | putative physarolisin |

List of genes differentially regulated in the presence of LAI-1 in comparison to control cells after 3 h of treatment. Differentially regulated genes were identified using the SAM program. The genes listed were more than 1.5 fold up- or downregulated in comparison to untreated cells. DDB_G ID is the dictyBase (http://dictybase.org/) gene identification number. The gene name and the gene product description were obtained from dictyBase. FC: Fold Change.
