# Supplementary material for: Inter-kingdom Signaling by the Legionella Quorum Sensing Molecule LAI-1 Modulates Cell Migration through an IQGAP1-Cdc42-ARHGEF9-Dependent Pathway
Source: PLoS Pathog. 2015 Dec 3;11(12):e1005307. doi: 10.1371/journal.ppat.1005307 (PMC4669118; doi:10.1371/journal.ppat.1005307)
Supplement: S3 Table — (DOCX) [file ppat.1005307.s012.docx]

**Table S3. Oligonucleotides used for RNA interference.**

| **NCBI gene** | **Gene description** | **Entrez gene ID** | **Product name** | **Product ID** |
| --- | --- | --- | --- | --- |
| ARHGAP1 | Rho GTPase activating protein 1 | 392 | Hs_ARHGAP1_5 | SI03233797 |
|  |  |  | Hs_ARHGAP1_6 | SI04144126 |
|  |  |  | Hs_ARHGAP1_7 | SI0416481 |
|  |  |  | Hs_ARHGAP1_8 | SI04177754 |
| ARHGAP17 | Rho GTPase activating protein 17 | 70497 | Hs_ARHGAP17_1 | SI00302001 |
|  |  |  | Hs_ARHGAP17_3 | SI00302036 |
|  |  |  | Hs_ARHGAP17_4 | SI00302043 |
|  |  |  | Hs_ARHGAP17_5 | SI02780449 |
| ARHGEF9 | Cdc42 guanine nucleotide | 23229 | Hs_ARHGEF9_5 | SI04138498 |
|  | exchange factor |  | Hs_ARHGEF9_7 | SI04210689 |
|  |  |  | Hs_ARHGEF9_10 | SI05428654 |
|  |  |  | Hs_ARHGEF9_11 | SI05428661 |
| CD2AP | CD2-associated protein | 23607 | Hs_CD2AP_1 | SI00097314 |
|  |  |  | Hs_CD2AP_2 | SI00097321 |
|  |  |  | Hs_CD2AP_7 | SI03033681 |
|  |  |  | Hs_CD2AP_8 | SI03110884 |
| Cdc42 | cell division cycle 42 | 998 | Hs_CDC42_4 | SI00028413 |
|  |  |  | Hs_CDC42_7 | SI02757328 |
|  |  |  | Hs_CDC42_15 | SI04381671 |
|  |  |  | Hs_CDC42_17 | SI04948440 |
| DOCK11 | dedicator of cytokinesis 11 | 139818 | Hs_DOCK11_5 | SI04157202 |
|  |  |  | Hs_DOCK11_6 | SI04257743 |
|  |  |  | Hs_DOCK11_7 | SI04277035 |
|  |  |  | Hs_DOCK11_8 | SI04330795 |
| FGD1 | FYVE, RhoGEF and | 2245 | Hs_FGD1_2 | SI00386568 |
|  | PH domain containing 1 |  | Hs_FGD1_5 | SI03170818 |
|  |  |  | Hs_FGD1_7 | SI04203192 |
|  |  |  | Hs_FGD1_9 | SI04280087 |
| IQGAP1 | IQ motif containing | 8826 | Hs_IQGAP1_1 | SI00057036 |
|  | GTPase activating protein 1 |  | Hs_IQGAP1_2 | SI00057043 |
|  |  |  | Hs_IQGAP1_3 | SI00057050 |
|  |  |  | Hs_IQGAP1_5 | SI02655268 |
| Rac1 | ras-related C3 | 5879 | Hs_RAC1_5 | SI02638293 |
|  | botulinum toxin substrate 1 |  | Hs_RAC1_6 | SI02655051 |
|  |  |  | Hs_RAC1_7 | SI03037524 |
|  |  |  | Hs_RAC1_8 | SI03040884 |

| Ran | RAN, member RAS oncogene family | 5901 | Hs_RAN_7 | SI04950498 |
| --- | --- | --- | --- | --- |
|  |  |  | Hs_RAN_8 | SI04950505 |
|  |  |  | Hs_RAN_9 | SI04950512 |
|  |  |  | Hs_RAN_10 | SI04950519 |
| RanBP1 | RAN binding protein 1 | 5902 | Hs_RANBP1_3 | SI00698201 |
|  |  |  | Hs_RANBP1_4 | SI00698208 |
|  |  |  | Hs_RANBP1_6 | SI03188381 |
|  |  |  | Hs_RANBP1_7 | SI04142089 |
| RhoA | ras homolog family member A | 387 | Hs_RHOA_1 | SI00702695 |
|  |  |  | Hs_RHOA_6 | SI02654211 |
|  |  |  | Hs_RHOA_7 | SI02654267 |
|  |  |  | Hs_RHOA_8 | SI02776907 |
