# Supplementary material for: Inter-kingdom Signaling by the Legionella Quorum Sensing Molecule LAI-1 Modulates Cell Migration through an IQGAP1-Cdc42-ARHGEF9-Dependent Pathway
Source: PLoS Pathog. 2015 Dec 3;11(12):e1005307. doi: 10.1371/journal.ppat.1005307 (PMC4669118; doi:10.1371/journal.ppat.1005307)
Supplement: S4 Table — (DOCX) [file ppat.1005307.s013.docx]

**Table S4. Primer pairs used for quantitative real-time PCR analysis.**

| **dictyBase ID** | **Gene name** | **Sequence** |
| --- | --- | --- |
| DDB_G0275689 | *abcG2* | Forward: 5'- GCGTTCACTTTTCTTGGGTG -3'  Reverse: 5'- TTGCGAAAACCATTGCCAAC -3' |
| DDB_G0288065 | *cdcD* | Forward: 5'- GCACGTTCTAGAGGTTCATCAC -3'  Reverse: 5'- GCACCAGAGAAACCTTGAGTCT -3' |
| DDB_G0286191 | *atg8* | Forward: 5'- ATGGTTCATGTATCAAGCTTTA -3'  Reverse: 5'- TTATAAATCACTACCAAAAGTATT -3' |
| DDB_G0272783 | *rliA* | Forward: 5'- TGGTCAAGATATCTGGTCAACC -3'  Reverse: 5'- TCCTTGAACTTCTTGAGGTGTAG -3' |
| DDB_G0282559 | *dduA* | Forward: 5'- TCGAGTGAACATGACTATTTCCC -3'  Reverse: 5'- TTCAACATCGCCACCAGTAC -3' |
| DDB_G0293850 | *alrA* | Forward: 5'- TGAAGTTGCTCTCGATGCTG -3'  Reverse: 5'- TTAACGAGGCCAGCATCAAC -3' |
