# Supplementary material for: Inter-kingdom Signaling by the Legionella Quorum Sensing Molecule LAI-1 Modulates Cell Migration through an IQGAP1-Cdc42-ARHGEF9-Dependent Pathway
Source: PLoS Pathog. 2015 Dec 3;11(12):e1005307. doi: 10.1371/journal.ppat.1005307 (PMC4669118; doi:10.1371/journal.ppat.1005307)
Supplement: S1 Text — (DOCX) [file ppat.1005307.s014.docx]

# Chemical Synthesis

# General Experimental Section

All reactions were performed under an atmosphere of argon and in oven-dried glassware (200 °C oven temperature) unless specified otherwise. Tetrahydrofuran (THF) and diethyl ether (Et_2_O) was distilled prior to use from sodium and benzophenone. *N*,*N*-dimethylformamide (DMF) and dichloromethane (CH_2_Cl_2_) purchased from *Acros Organics* as 'extra dry' reagents under inert gas atmosphere and over molecular sieves. All other reagents were purchased from commercial sources and were used without further purification. Petroleum ether (PE) refers to fractions of isohexanes, which boil between 40 and 80 °C.

Analytical thin-layer chromatography (TLC) was carried out using pre-coated glass plates (silica gel 60 F_254_) from *Merck*, and visualized by exposure to ultraviolet light (UV, 254 nm) and by staining with aqueous acidic ceric ammonium molybdate(IV) (CAM) or potassium permanganate solution. Flash column chromatography was performed using *Merck* silica gel 60 (40-63 µm particle size).

Proton nuclear magnetic resonance (^1^H-NMR) spectra were recorded on a Varian 400, Inova 400 or Varian 600 spectrometer. Chemical shifts (δ scale) are expressed in parts per million (ppm) and are calibrated using residual protic solvent as an internal reference. Data for ^1^H-NMR spectra are reported as follows: chemical shift (δ ppm) (multiplicity, coupling constants (Hz), integration). Couplings are expressed as: s = singlet, d = doublet, t = triplet, q = quartet, m = multiplet, br = broad, or combinations thereof. Carbon nuclear magnetic resonance (^13^C-NMR) spectra were recorded on the same spectrometers at 75, 100 and 150 Hz, respectively. Carbon chemical shifts (δ scale) are also expressed in parts per million (ppm) and are referenced to the central carbon resonances of the solvents (CDCl_3_: δ = 77.16 ppm).^[^[^1^](#_ENREF_1)^]^

Infrared (IR) spectra were recorded on a Perkin Elmer Spectrum BX II (FTIR System) equipped with an attenuated total reflection (ATR) measuring unit. IR data is reported in frequency of absorption (cm^‒1^). The IR bands are characterized as: w = weak, m = medium, s = strong, br = broad, or combinations thereof.

Mass spectroscopy (MS) experiments were performed on a Thermo Finnigan MAT 95 (electron ionization, EI) or on a Thermo Finnigan LTQ FT (electrospray ionization, ESI) instrument.

Melting points (mp) were measured on a Büchi Melting Point B-540 or SRS MPA120 EZ-Melt apparatus and are uncorrected.

Optical rotations were measured at the given temperature (T in [°C]) on a Perkin-Elmer 241 or Krüss P8000-T polarimeter using a sodium lamp (λ = 589 nm, d-line). Measurements were carried out in a cell with a path length (l) of 0.5 dm. Concentrations (*c*) are expressed in g/(100 mL). Specific rotations ${([\alpha]}_{D}^{T}$) were calculated using the equation ${[\alpha]}_{D}^{T}$ = 100·α/(*c*·l) and are reported in 10^‒1^ deg cm^2^ g^‒1^.

High performance liquid chromatography (HPLC) was performed with HPLC grade solvents and deionized water that was purified on a TKA MicroPure water purification system. All solvents were degassed with helium gas prior to use. All experiments were carried out at room temperature; the column used is specified as appropriate.

Intermediate weinreb amides **3** ^[1]^ and **7** ^[2]^ were prepared according to reported literature protocols and obtained in comparable yields.

# Experimental Procedures

(3*S*)-3-(2,2-dimethyl-1,1-diphenylpropoxy)pentadecan-4-one (4)

Magnesium turnings (61.9 mg, 2.55 mmol, 4.9 eq) were dried at 650°C (heat gun) *in vacuo* (10^-3^ mbar) for 10 min. After cooling to room temperature, Et_2_O (0.5 mL) and 1,2-dibromoethane (6.79 µL, 78.8 µmol, 20 mol%) were added. A solution of 1-bromoundecane (470 µL, 2.11 mmol, 4.1 eq) in Et_2_O (2 mL) was added dropwise over a period of 15 min, and the resulting mixture was stirred at room temperature for an additional 30 min. This freshly prepared Grignard solution was added dropwise to a 0°C cold solution of Weinreb amide **3** ^[1]^ (200 mg, 0.519 mmol, 1.0 eq) in Et_2_O (5 mL). The reaction mixture was stirred at 0 °C for 1 h, then allowed to warm to room temperature and stirred at this temperature for an additional 21 h. The mixture was diluted with Et_2_O (10 mL) and the reaction quenched at 0°C with water (10 mL). The mixture was allowed to warm to room temperature, the organic phase was separated and washed with aq. KHSO_4_ (10 wt %) and brine, then dried over MgSO_4_ and concentrated *in vacuo*. Flash column chromatography [PE:EA 100:0→49:1→19:1] afforded ketone **4** (94.2 mg, 0.200 mmol, 38%) as a colorless liquid.

R*_f_* = 0.50 [PE:EtOAc 19:1].

${[\alpha]}_{D}^{21}$ = ‒21.6 (*c* = 0.75, CHCl_3_).

^1^H-NMR (600 MHz, CDCl_3_): δ = 7.66 – 7.59 (m, 4H), 7.45 – 7.40 (m, 2H), 7.39 – 7.33 (m, 4H), 4.11 (dd, *J* = 6.2, 5.4 Hz, 1H), 2.44 (ddd, *J* = 17.8, 8.4, 6.3 Hz, 1H), 2.35 (ddd, *J* = 17.8, 8.4, 6.4 Hz, 1H), 1.68 – 1.56 (m, 2H), 1.45 – 1.34 (m, 2H), 1.33 – 1.14 (m, 16H), 1.11 (s, 9H), 0.88 (t, *J* = 7.1 Hz, 3H), 0.81 (t, *J* = 7.5 Hz, 3H) ppm.

^13^C-NMR (150 MHz, CDCl_3_): δ = 213.04, 135.99, 135.95, 133.82, 133.36, 129.99, 129.96, 127.83, 127.77, 80.16, 38.16, 32.07, 29.78, 29.77, 29.62, 29.58, 29.50, 29.39, 27.96, 27.16, 23.10, 22.85, 19.54, 14.28, 8.96 ppm.

IR (ATR): *ṽ* = 2925 (m), 2855 (m), 1716 (w), 1428 (w), 1106 (m), 700 (s) cm^‒1^.

HRMS (EI): calcd. for C_30_H_45_O_2_Si^+^: 465.3183 [M−CH_3_]^+^ found: 465.3184 [M−CH_3_]^+^.

(3*S*)-3-hydroxypentadecan-4-one (5, (S)-LAI-1)

A solution of silyl ether **4** (128 mg, 0.266 mmol, 1.0 eq) in THF (4.5 mL) was cooled to 0°C and a solution of tetrabutylammonium fluoride in THF (1.0 M, 772 µL, 0.772 mmol, 2.9 eq) was added dropwise. The resulting mixture was allowed to warm to room temperature and stirred at this temperature for 3 h. The reaction was quenched with aq. NaHCO_3_ (15 mL of a saturated solution) and extracted with Et_2_O (2 x 20 mL). The combined organic fractions were washed with water, dried over MgSO_4_ and concentrated *in vacuo*. Flash column chromatography [PE:EtOAc 100:0→19:1] and further purification by HPLC [Dynamax Microsorb 60-8 C18 (250 x 21.4 mm), isocratic elution, water (A)/MeCN (B); 78% B; flow rate 16.8 mL/min; detection 200 nm: t_R_ (**5**) = 27.3 min] afforded LAI-1 (**5**) (36.2 mg, 0.149 mmol, 56%) as a colorless liquid.

R*_f_* = 0.43 [PE:EtOAc 9:1].

${[\alpha]}_{D}^{21}$ = ‒51.6 (*c* = 0.90, CHCl_3_).

^1^H-NMR (400 MHz, CDCl_3_): δ = 4.15 (dd, *J* = 6.8, 4.0 Hz, 1H), 3.44 (br s, 1H), 2.50 – 2.38 (m, 2H), 1.96 – 1.83 (m, 1H), 1.66 – 1.52 (m, 3H), 1.34 – 1.17 (m, 16H), 0.93 (t, *J* = 7.4 Hz, 3H), 0.87 (t, *J* = 6.9 Hz, 3H) ppm.

^13^C-NMR (100 MHz, CDCl_3_): δ = 212.58, 77.30, 38.03, 32.05, 29.73, 29.73, 29.58, 29.49, 29.47, 29.39, 26.90, 23.76, 22.83, 14.26, 9.02 ppm.

IR (ATR): *ṽ* = 3482 (br, w), 2923 (s), 2854 (m), 1710 (s), 1464 (m), 1075 (w), 981 (m), 722 (w) cm^‒1^.

HRMS (EI): calcd. for C_15_H_30_ClO_2_^−^: 277.1940 [M+Cl]^−^ found: 277.1936 [M+Cl]^−^.

(3*S*)-3-(*tert*-butoxycarbonyl)aminopentadecan-4-one (8)

To magnesium turnings (167 mg, 6.91 mmol, 10 eq) in THF (1 mL) was added a solution of 1-bromoundecane (1.46 mL, 6.56 mmol, 9.5 eq) in THF (4 mL) dropwise over a period of 15 min, and the resulting mixture was stirred at 50 ºC for additional 1 h. This freshly prepared Grignard solution was added dropwise to a solution of Weinreb amide **7** ^[2]^ (170 mg, 0.69 mmol, 1.0 eq) in THF (5 mL) at 0°C. The reaction mixture was stirred at room temperature for 2 h. The reaction was diluted with Et_2_O (20 mL) and then quenched at 0°C with saturated NH_4_Cl (10 mL). The mixture was allowed to warm to room temperature, the organic phase was separated and washed with satd. NH_4_Cl and brine, then dried over MgSO_4_ and concentrated *in vacuo*. Flash column chromatography [hexane:EA 98:2→95:5] yielded ketone **8** (110 mg, 0.32 mmol, 47%) as a colorless oil.

R*_f_* = 0.52 [hexane:EA 85:15].

${[\alpha]}_{D}^{20}$ = 42.2 (*c* = 1.0, CHCl_3_).

^1^H NMR (400 MHz, CDCl_3_): δ 5.24 (d, *J* = 7.6 Hz, 1H), 4.30 – 4.22 (m, 1H), 2.48 – 2.41 (m, 2H), 1.95 – 1.81 (m, 1H), 1.63 – 1.50 (m, 3H), 1.41 (s, 9H), 1.29 – 1.16 (m, 16H), 0.85 (t, *J* = 7.0 Hz, 6H) ppm.

^13^C NMR (100 MHz, CDCl_3_): δ 209.61, 155.57, 79.64, 60.25, 39.92, 32.01, 29.70, 29.55, 29.48, 29.43, 29.32, 28.44, 24.91, 23.63, 22.79, 14.21, 9.33 ppm.

HRMS (ESI): calcd. for C_40_H_78_N_2_NaO_6_^+^: 705.5758 [2M+Na] ^+^ found: 705.5780 [2M+Na] ^+^

(3*S*)-3-aminopentadecan-4-one hydrochloride (9, (S)-amino-LAI-1)

Ketone **8** (40 mg, 0.12 mmol) was dissolved in 2 M HCl in Et_2_O (5 mL) and stirred for 48 h at room temperature. The reaction mixture was evaporated to dryness and the white precipitate was recrystallized from CHCl_3_ and Et_2_O, yielding (S)-amino-LAI-1 **9** as fine white, waxy crystals (24 mg, 83%).

R*_f_* = 0.21 [CH_2_Cl_2_:MeOH 95:5].

${[\alpha]}_{D}^{20}$ = 22.4 (*c* = 1.0, MeOH).

mp: 126 °C (sharp – no interval)

^1^H NMR (400 MHz, CD_3_OD): δ 4.13 (dd, *J* = 7.2, 4.3 Hz, 1H), 2.71 – 2.52 (m, 2H), 2.15 – 2.00 (m, 1H), 1.98 – 1.82 (m, 1H), 1.68 – 1.55 (m, 2H), 1.39 – 1.22 (m, 16H), 1.01 (t, *J* = 7.5 Hz, 3H), 0.90 (t, *J* = 6.8 Hz, 3H) ppm.

^13^C NMR (100 MHz, MeOD): δ 207.12, 60.97, 39.82, 33.06, 30.73, 30.71, 30.59, 30.53, 30.46, 30.13, 24.27, 23.94, 23.73, 14.44, 9.27 ppm.

HRMS (ESI): calcd. for C_30_H_62_N_2_NaO_2_^+^: 505.4709 [2M+Na] ^+^ found: 505.4761 [2M+Na] ^+^

# Appendices

## NMR Spectra

CDCl_3_, 600 MHz

CDCl_3_, 150 MHz

CDCl_3_, 600 MHz

CDCl_3_, 100 MHz

CDCl_3_, 400 MHz

CDCl_3_, 100 MHz

^^

^^

CD_3_OD, 400 MHz

^^

CD_3_OD, 100 MHz

# Literature

[1] D. A. Higgins, M. E. Pomianek, C. M. Kraml, R. K. Taylor, M. F. Semmelhack, B. L. Bassler, *Nature* **2007**, 450, 883−886.

[2] A. Johansson, A. Poliakov, E. Akerblom, K. Wiklund, G. Lindeberg, S. Winiwarter, U. H. Danielson, B. Samuelsson, A. Hallberg, *Bioorg. Med. Chem.* **2003**, 11(12), 2551-2558.
